# Supplementary material for: MalDeepSeq panel: A targeted ultra-deep sequencing approach to trace drug resistance markers in Plasmodium falciparum
Source: Cell Rep Methods. 2026 Jun 25;6(9):101509. doi: 10.1016/j.crmeth.2026.101509 (PMC13615499; doi:10.1016/j.crmeth.2026.101509)
Supplement: Supplementary file 1 [file mmc1.pdf]

Cell Reports Methods, Volume *6*

Supplemental information

MalDeepSeq panel: A targeted ultra-deep sequencing approach to trace drug resistance markers in *Plasmodium falciparum*

Yanka E.A. R. Salazar, Antonio M. Rezende, Maria C.S. B. Puça, Sonja Lagström, Danielle Fletcher, Julian Muwanguzi-Karugaba, Colin J. Sutherland, Khalid B. Beshir, Maria E.P. Mascarenhas, Jaime Louzada, Joseli Oliveira-Ferreira, Abdoulaye Djimde, Dinora Lopes, Tais N. de Sousa, and José P. Gil

SUPPLEMENTAL FIGURES

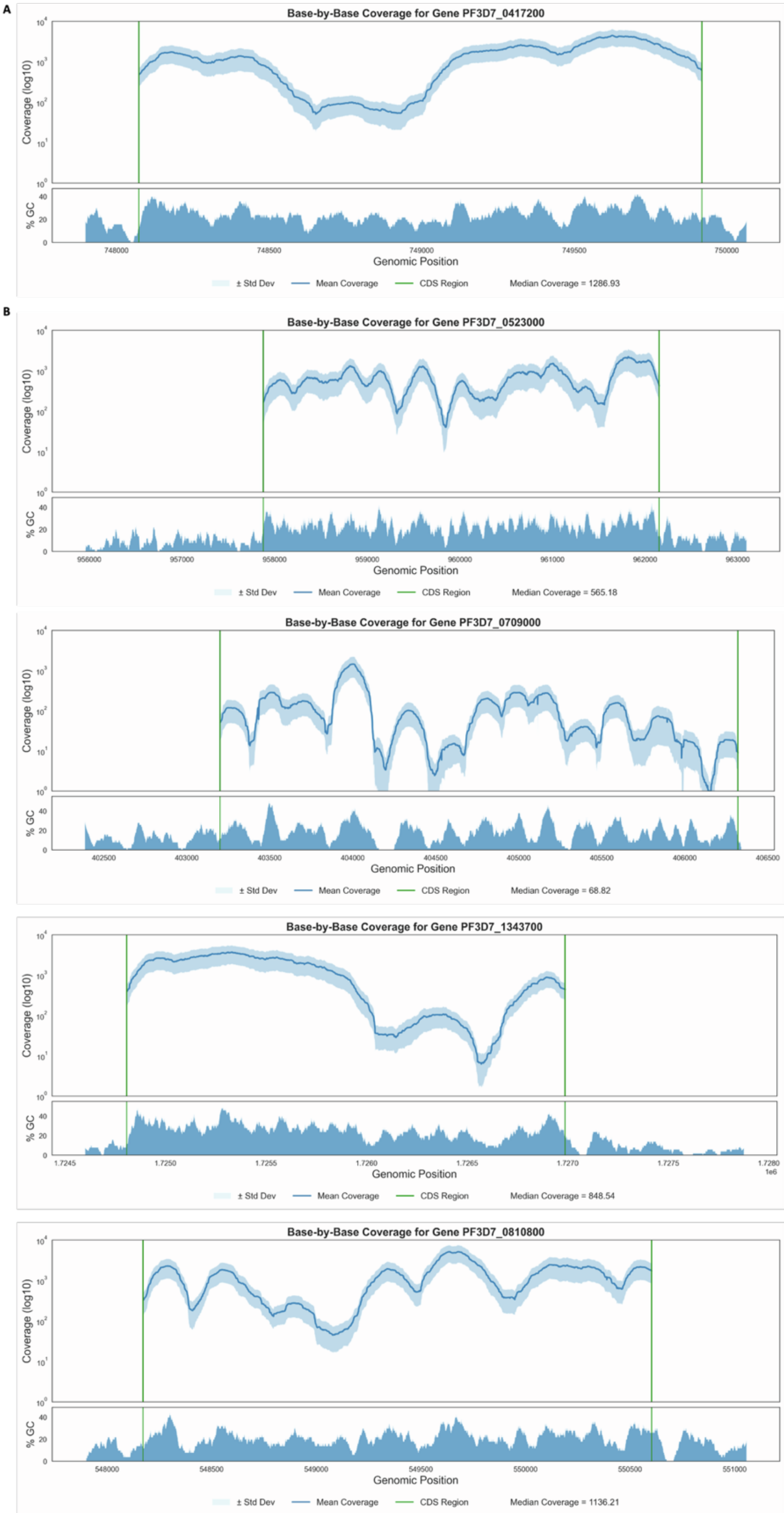

Figure S1. Per-base read depth and GC-content across target genes, related to Figure 2. Panels A–E show per-base read depth for each gene: A = *pfdhfr*, B = *pfmdr1*, C = *pfcrt*, D = *pfk13*, E = *pfdhps*. The solid line represents the mean read depth across samples at each genomic position, and the shaded band corresponds to ±1 SD. The CDS median read depth is reported in the legend as the median of the position-wise mean depth restricted to the CDS. Vertical lines mark CDS boundaries. The lower panel for each gene displays the GC-content (%) computed using a centered sliding window (50 bp), allowing visualization of GC variability across the region. X-axis: genomic coordinates fixed to the exact gene interval (start–end).

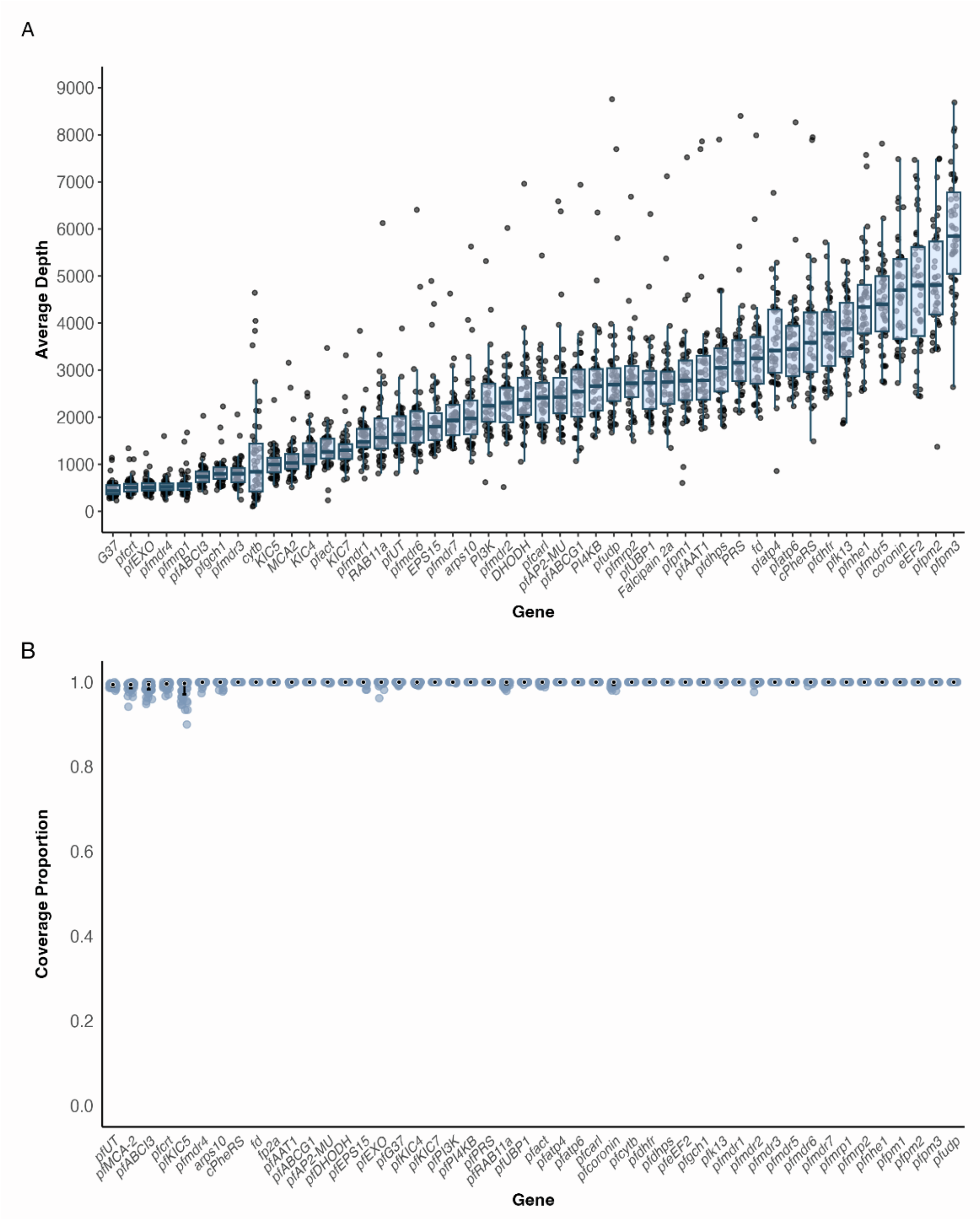

Figure S2. Sequencing depth and coverage proportion across the 48-gene panel in the WB group (Brazil), related to Figure 2. A) Average read depth per gene within the coding sequence (CDS), ordered by median depth. B) CDS coverage proportion (breadth) for the same genes. Overall performance was uniform, with consistently high depth and coverage across loci. The lowest median depth was observed for *G37* (430.96×).

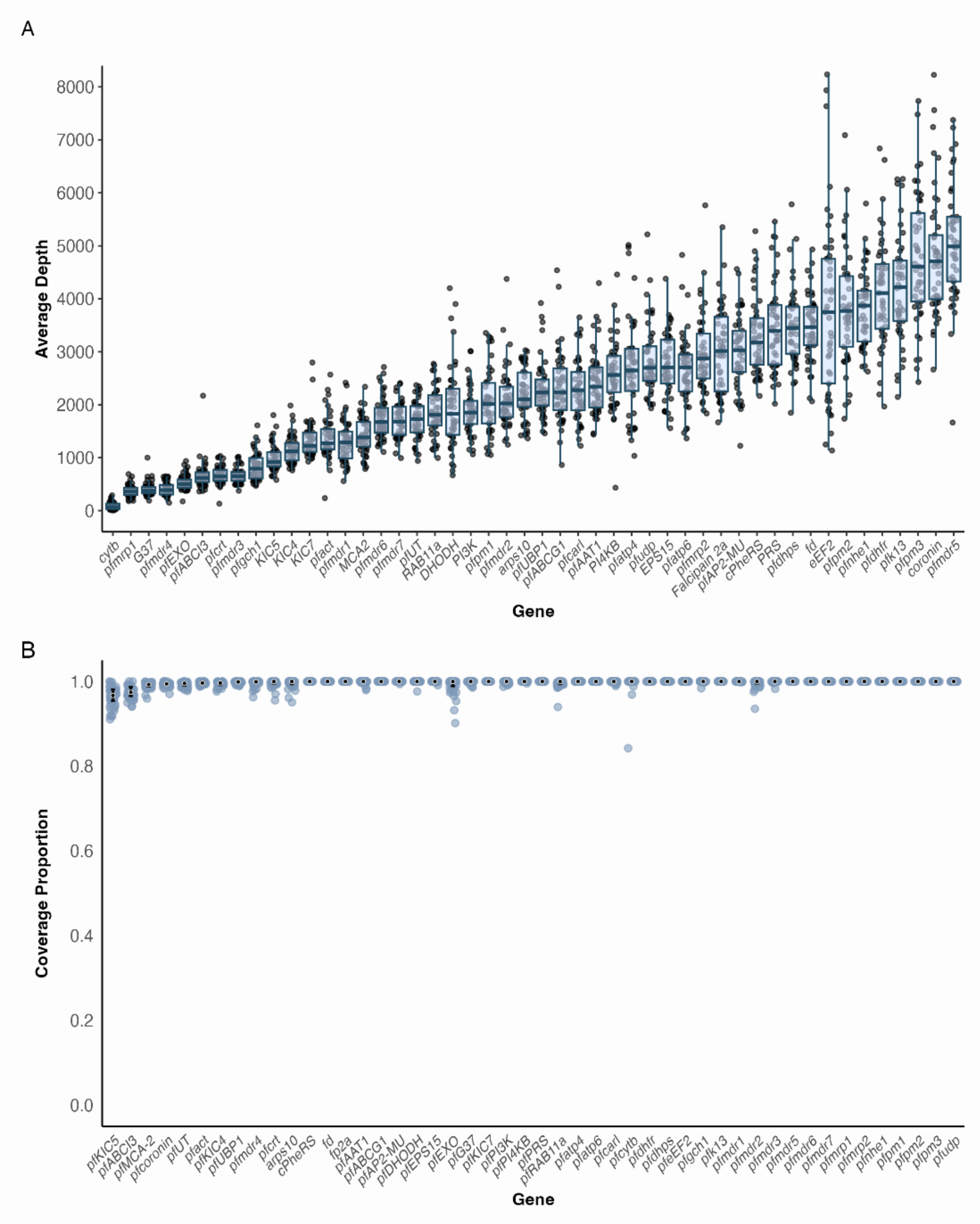

Figure S3. Sequencing depth and coverage proportion across the 48-gene panel in the DBS group (Mali), related to Figure 2. (A) Average CDS read depth and (B) CDS coverage proportion for the DBS group. The lowest median depth was 66.9× for *cytb*.

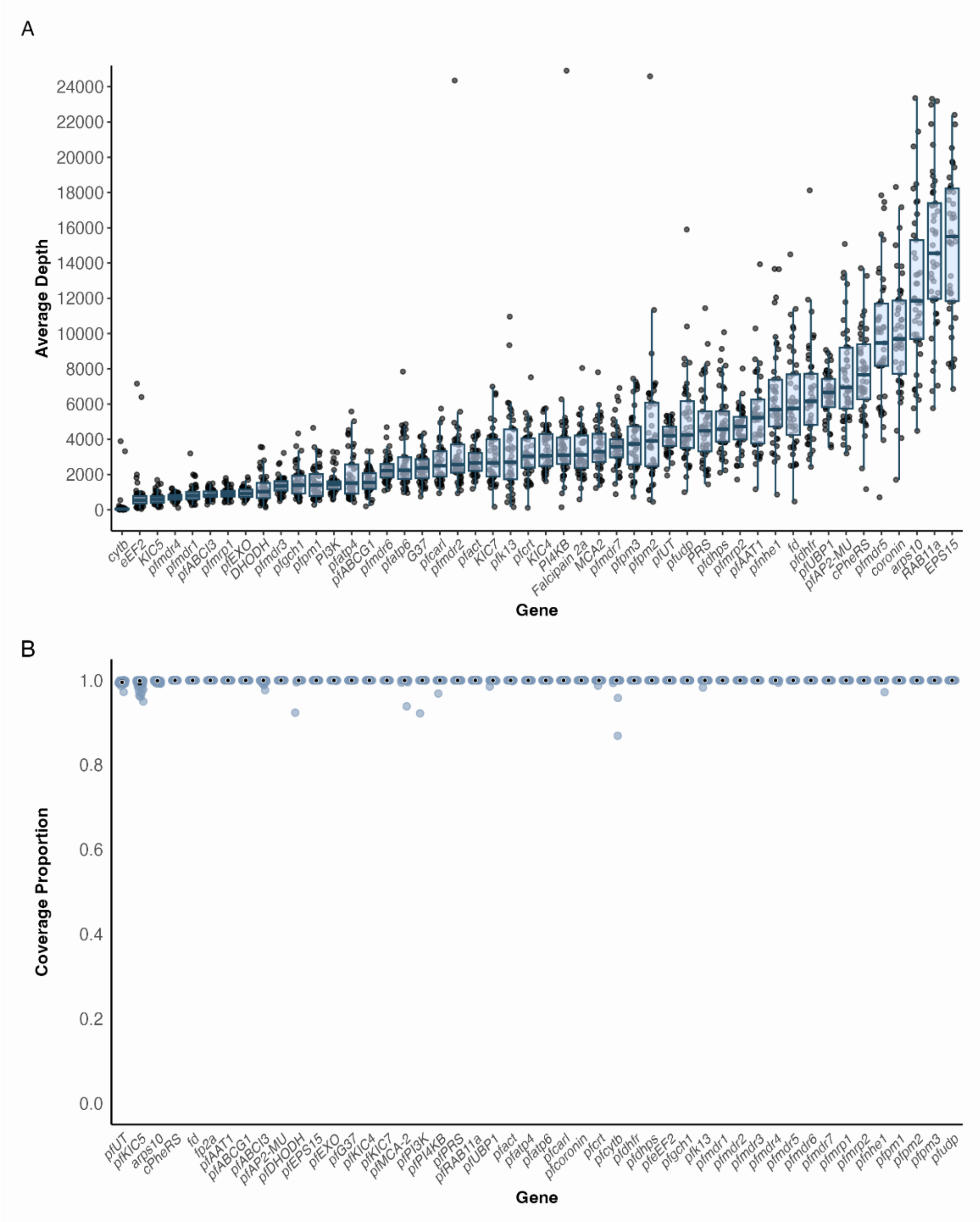

Figure S4. Sequencing depth and coverage proportion across the 48-gene panel in the DBS-opt group (São Tomé and Príncipe), related to Figure 2. A) Average CDS read depth and B) CDS coverage proportion. This subset exhibited the widest depth variability, with some loci reaching up to 24,000×, indicating efficient amplification despite limited starting material. The adapted workflow, including modified SWGA cleanup and pooling, likely contributed to maintaining robust coverage and overall high performance under low-DNA conditions. The lowest median depth was 36.3× for *cytb*.

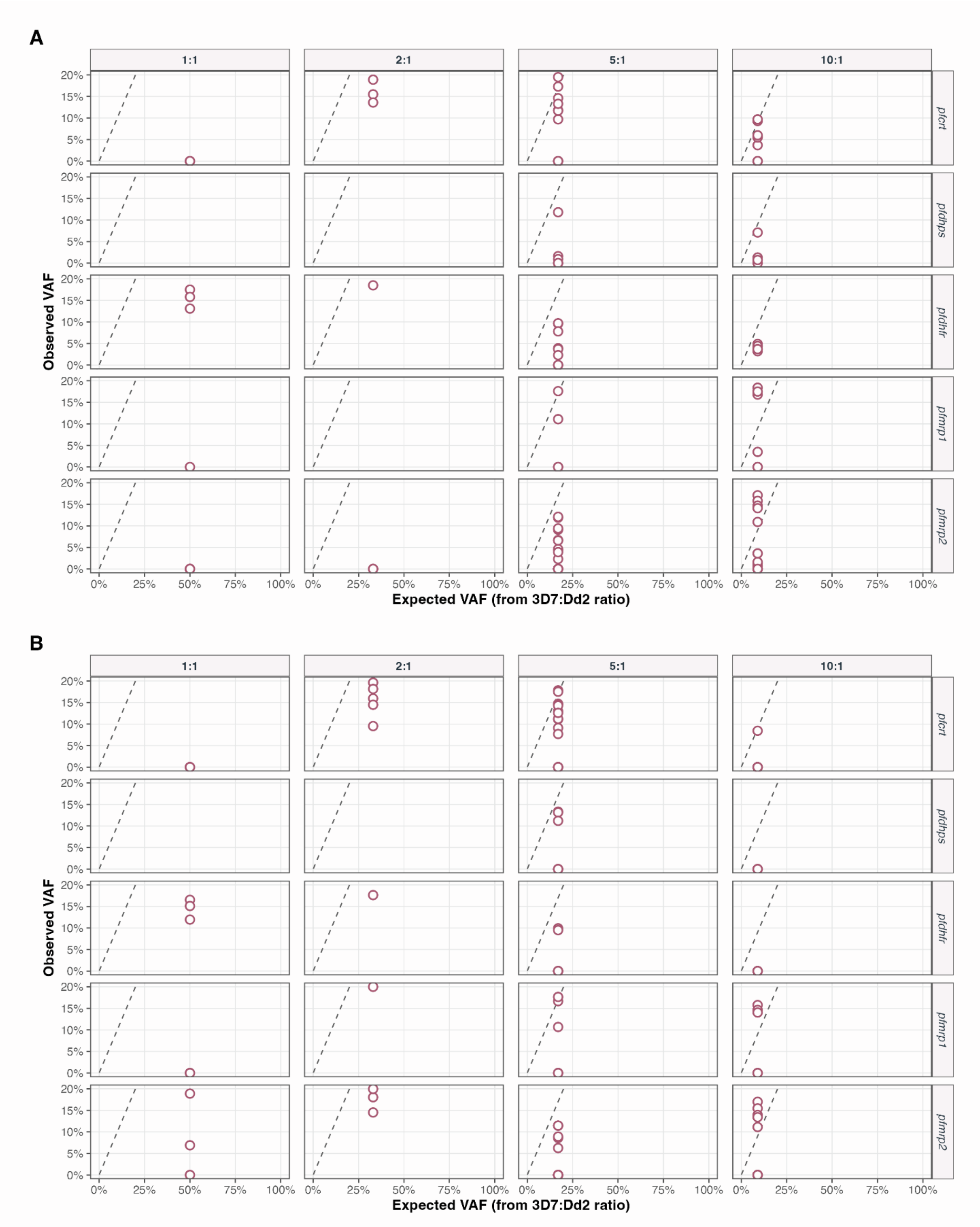

Figure S5. Zoomed view of observed versus expected variant allele frequencies (VAFs) in the low-frequency range (0–20%), related to Figure 3. (A) Mutect2 and (B) HaplotypeCaller. Points represent Dd2-specific loci across 3D7:Dd2 mixtures, stratified by gene and dilution ratio. The dashed line indicates the expected VAF. Compared with the full-range visualization, this zoom highlights reduced dispersion and improved agreement between observed and expected values in more asymmetric mixtures (5:1 and 10:1), while maintaining locus-specific variability.

SUPPLEMENTAL TABLES

Table S1. Genes included in the 48-target *Plasmodium falciparum* target enrichment panel related to the STAR Methods section.

Name	Gene size (nt)	Denomination	Chr Position (Start)	Chr Position (End)
pfcrt	3957	PF3D7_0709000	402385	406341
pfk13	3278	PF3D7_1343700	1724600	1727877
fpa2	3670	PF3D7_1115700	591136	594805
pfdhfr	2169	PF3D7_0417200	747897	750065
pfdhps	3162	PF3D7_0810800	547896	551057
pfcarl	6843	PF3D7_0321900	921776	928618
pfudp	2003	PF3D7_1113300	518688	520690
pfact	3854	PF3D7_1036800	1447919	1451772
cytb	1131	PF3D7_MIT02300	3492	4622
pfatp4	6137	PF3D7_1211900	527829	533965
pfatp6	5748	PF3D7_0106300	264487	270234
pfpm1	4162	PF3D7_1407900	287116	291277
pfpm2	3018	PF3D7_1408000	292244	295261
pfpm3	2419	PF3D7_1408100	296683	299101
pfEXO	3146	PF3D7_1362500	2502730	2505875
pfAAT1	4212	PF3D7_0629500	1213102	1217313
pfUT	15765	PF3D7_0704600	214949	230713
pfnhe1	8676	PF3D7_1303500	169481	178156
pfAP2-MU	3966	PF3D7_1218300	716700	720665
pfUBP1	12905	PF3D7_0104300	188420	201324
pfgch1	1872	PF3D7_1224000	974226	976097
pfmdr1	7141	PF3D7_0523000	955955	963095
pfmdr2	5674	PF3D7_1447900	1953474	1959147
pfmdr3	3335	PF3D7_1145500	1807031	1810365
pfmdr4	4821	PF3D7_0302600	141335	146155
pfmdr5	3457	PF3D7_1339900	1598111	1601567
pfmdr6	4359	PF3D7_1352100	2075535	2079893
pfmdr7	3247	PF3D7_1209900	448322	451568
pfmrp1	8128	PF3D7_0112200	463764	471891
pfmrp2	10802	PF3D7_1229100	1192185	1202986
pfABCI3	11052	PF3D7_0319700	820232	831283
pfABCG1	2344	PF3D7_1426500	1027682	1030025
fd	1256	PF3D7_1318100	748160	749415
arps10	2169	PF3D7_1460900	2480089	2482257
pfPI3K	6629	PF3D7_0515300	628784	635412
pfcPheRS	575	PF3D7_0109800	380254	382568
pfPI4KB	1559	PF3D7_0509800	409403	415758
pfDHODH	569	PF3D7_0603300	130191	132792
pfKIC7	599	PF3D7_0813000	643066	645158
pfEPS15	980	PF3D7_1025000	1045271	1051309
pfKIC5	1870	PF3D7_1138700	1525654	1532527
pfG37	349	PF3D7_1204400	208165	210893

pfPRS	746	PF3D7_1213800	589729	593777
pfKIC4	888	PF3D7_1246300	1925058	1929618
pfcoronin	602	PF3D7_1251200	2091731	2094720
pfRAB11a	216	PF3D7_1320600	845865	848770
pfMCA2	2020	PF3D7_1438400	1548072	1557053
pfeEF2	832	PF3D7_1451100	2089730	2094221

The 48 genes included in this panel were selected to represent three complementary categories: (i) well-established molecular markers of antimalarial resistance (e.g., *pfcrt, pfmdr1, pfk13, pfdhfr, pfdhps*); (ii) genes involved in drug transport, metabolism, or parasite fitness, potentially influencing treatment response; and (iii) emerging or putative loci identified in recent genomic, experimental, or functional studies. This design aims to capture both known resistance determinants and additional variation that may contribute to evolving resistance phenotypes.

Table S2. Summary of clinical sample cohorts included in the study related to the STAR Methods section.

Cohort	Study type	Country	Sample type	Parasitemia (parasites/µL), median [IQR]	Library concentration (ng/µL), median [IQR]	Treatment context
**WB**	Cross-sectional	Brazil	Whole blood	9,240 [5,470–20,100]	5.0 [3.94–6.65]	Artemether-Lumefantrine (first-line) with a single low-dose of Primaquine
**DBS**	Clinical Trial	Mali	Dried blood spots	5,958 [1,828–12,596]	2.7 [1.80–3.17]	Artemether-Lumefantrine (first-line); Ganaplacide-Lumefantrine (Phase-2b study)
**DBS-opt**	Cross-sectional	São Tomé and Príncipe	Dried blood spots	9,600 [3,200–17,620]	0.5 [0.27–0.70]	Artesunate-Amodiaquine (first-line)

WB, whole blood; DBS, dried blood spots; DBS-opt, optimized DBS protocol. Parasitemia values were estimated by qPCR for DBS samples and routine methods for WB samples, as described in the Methods.

Table S3. Panel performance at 10×, 30× and 80× by subset of samples related to the STAR Methods section.

Sample set	Median depth (all loci)[a]	Successful loci[b] (%) @10×	Successful loci (%) @30×	Successful loci (%) @80×
**WB group**	698	100.0	91.7	72.9
**DBS group**	550	97.9	85.4	58.3
**DBS-opt group**	860	100.0	95.8	85.4
**Mock infections (1000 parasites)**	385	93.8	78.1	51.0
**Mock infections (100 parasites)**	231	81.2	62.5	39.6
**Mock infections (10 parasites)**	49	52.1	35.4	22.9

[a] Median depth (all loci) is the median per-base coverage across all CDS positions, irrespective of pass/fail status

[b] Successful loci (%) is the percentage of target coding sequences (CDS) whose breadth (fraction of CDS bases covered) is ≥80% at the indicated depth cutoff (10×, 30×, or 80×).

Table S4. Logistic model of CDS success versus parasite input in mock infections related to the STAR Methods section.

Depth threshold	Intercept (b0)	Slope (b1)	LoD95 (95% CI)
**10×**	-1.32	1.44	905 (448–2,609)
**30×**	-1.41	0.97	32,946 (8,751–322,131)
**80×**	-1.78	0.67	11,794,634 (512,015–14,846,122)

N = 432 gene–sample rows (9 mock mixtures × 48 loci). LoD95 and 95% confidence intervals were estimated from a logistic regression of success (CDS breadth ≥80%) versus $\log_{10}$(parasite input), with 10,000 parametric draws from the coefficient covariance matrix to generate percentile intervals. LoD95 represents the total parasite count at which the fitted model predicts 95% success, reflecting analytical sensitivity rather than clinical parasitemia.

Table S5. Comparison of median sequencing depth (IQR) across *Plasmodium falciparum* target genes obtained from WB, DBS and DBS-opt samples, related to Figure 2.

Gene	WB group	DBS group	DBS-opt group
pfDHODH	2367.60 (2049.90–2839.69)	1825.88 (1431.58–2302.88)	1032.71 (664.58–1528.57)
pfEPS15	1800.66 (1512.83–2084.57)	2705.03 (2398.43–3230.72)	15498.80 (11837.67–18224.77)
fpa2	2748.27 (2284.55–2970.85)	3013.64 (2249.68–3658.70)	3113.57 (2369.54–4218.84)
pfG37	430.96 (359.98–557.19)	376.43 (329.54–448.33)	2379.16 (1779.87–2873.33)
pfKIC4	1187.34 (1003.34–1448.35)	1118.72 (947.12–1278.08)	3068.52 (2478.25–4303.29)
pfKIC5	994.39 (827.95–1137.66)	918.91 (831.05–1105.13)	597.45 (412.88–769.23)
pfKIC7	1300.46 (1111.42–1428.15)	1224.19 (1109.43–1476.53)	2664.10 (1889.44–3999.49)
pfMCA2	1026.59 (899.55–1218.96)	1382.31 (1206.02–1676.50)	3301.97 (2706.96–4314.90)
PI3K	2242.01 (1901.80–2715.66)	1851.88 (1634.44–2072.51)	1444.24 (1163.71–1616.18)
PI4KB	2661.78 (2142.59–3024.74)	2560.37 (2237.22–2926.81)	3100.47 (2595.26–4111.88)
PRS	3156.36 (2766.03–3636.64)	3395.95 (2754.61–3884.98)	4476.81 (3312.15–5581.88)
pfRAB11a	1567.26 (1335.83–1976.21)	1808.39 (1605.46–2181.53)	14554.05 (11968.63–17398.65)
arps10	1973.41 (1634.19–2355.64)	2104.18 (1956.40–2609.10)	11852.34 (9691.44–15297.82)
pfcPheRS	3582.49 (2962.83–4227.09)	3176.70 (2756.38–3635.00)	7656.09 (6270.50–9389.10)
pfcoronin	4700.10 (3659.60–5362.01)	4705.37 (3991.43–5202.65)	9699.64 (7718.10–11874.49)
cytb	840.74 (422.32–1438.61)	66.95 (30.14–134.04)	36.36 (20.15–71.44)
pfeEF2	4799.27 (3723.91–5617.53)	3745.10 (2401.78–4752.73)	538.92 (340.32–781.91)
fd	3251.35 (2710.52–3705.70)	3466.15 (3121.47–3845.39)	5754.33 (4246.29–7686.74)
pfAAT1	2783.61 (2372.01–3306.08)	2339.34 (1947.06–2707.46)	5227.25 (3758.77–6253.67)
pfABCG1	2545.43 (2012.14–3013.93)	2236.17 (1899.98–2685.72)	1555.14 (1187.27–2080.02)
pfABCI3	734.69 (623.15–846.98)	618.92 (538.32–725.66)	835.98 (729.73–1043.85)
pfAP2-MU	2428.31 (2083.36–2838.77)	3027.69 (2615.38–3397.81)	6947.46 (5743.98–9203.49)
pfEXO	510.85 (442.26–601.16)	503.20 (428.24–589.13)	920.14 (730.80–1149.21)
pfUBP1	2730.90 (2178.07–2984.21)	2232.20 (1985.65–2476.21)	6652.93 (5816.01–7432.69)
pfUT	1635.11 (1450.05–2021.21)	1726.70 (1476.33–1972.77)	4205.42 (3613.09–4713.58)
pfact	1264.10 (1122.08–1542.93)	1271.77 (1157.92–1538.49)	2660.29 (2206.22–3226.40)
pfatp4	3416.62 (2943.24–4288.41)	2645.60 (2258.32–3057.08)	1516.46 (909.96–2576.70)
pfatp6	3450.23 (2868.80–3941.83)	2706.05 (2252.27–2947.45)	2240.22 (1751.59–2980.97)
pfcarl	2415.58 (1884.32–2731.22)	2273.14 (1892.75–2615.81)	2499.13 (1879.33–3326.30)
pfcrt	504.95 (420.41–586.64)	645.54 (560.26–771.90)	3034.51 (2388.08–4063.86)
pfdhfr	3781.89 (3091.08–4240.46)	4108.51 (3433.83–4652.45)	6151.37 (4813.23–7718.97)
pfdhps	3047.67 (2544.67–3465.39)	3450.77 (2961.80–3854.90)	4569.59 (3859.98–5595.46)
pfgch1	796.15 (690.17–942.66)	792.40 (606.62–994.86)	1395.71 (920.22–1944.12)
pfk13	3873.47 (3283.36–4430.12)	4220.91 (3579.62–4721.01)	2699.21 (1725.46–4564.03)
pfmdr1	1476.22 (1359.83–1752.97)	1287.43 (986.97–1494.63)	803.28 (596.47–1009.81)
pfmdr2	2308.95 (1893.95–2628.70)	2022.73 (1765.90–2341.00)	2562.17 (2103.99–3710.30)
pfmdr3	800.15 (616.05–923.58)	648.91 (560.55–728.45)	1350.09 (1091.37–1623.84)
pfmdr4	518.32 (447.93–592.21)	388.15 (304.30–485.23)	704.20 (566.31–849.79)

pfmdr5	4400.85 (3823.06–4996.70)	4986.89 (4326.75–5548.00)	9464.73 (8165.24–11702.69)
pfmdr6	1758.14 (1458.25–2130.92)	1670.88 (1469.95–1938.16)	2223.21 (1830.14–2598.75)
pfmdr7	1932.09 (1614.83–2262.04)	1680.66 (1430.35–1962.87)	3570.18 (2970.61–3999.37)
pfmrp1	541.35 (454.33–597.08)	360.66 (296.52–433.15)	903.48 (750.96–1081.04)
pfmrp2	2720.11 (2425.11–3085.62)	2871.49 (2493.97–3341.49)	4727.88 (3989.99–5274.29)
pfnhe1	4339.80 (3778.31–4813.48)	3870.77 (3194.55–4154.62)	5681.82 (4722.25–7385.13)
pfpm1	2775.97 (2362.19–3204.55)	2017.80 (1647.09–2410.16)	1408.63 (776.27–2028.62)
pfpm2	4808.87 (4182.86–5734.76)	3769.97 (3095.04–4428.22)	3903.72 (2466.85–6081.70)
pfpm3	5847.36 (5046.01–6776.87)	4609.65 (3946.37–5619.44)	3738.68 (2570.14–4734.04)
pfudp	2691.28 (2342.42–3042.44)	2696.75 (2447.87–3102.28)	4240.64 (3503.83–6162.77)

Table S6. Global mixture quantification metrics by gene for Mutect2 and HaplotypeCaller, related to Figure 3.

Gene	Caller	Spearman ρ	Spearman p-value	Global bias (pp)	Global MAE (pp)	Wilcoxon p
pfcrt	HaplotypeCaller	0.583	3.73e-06	−9.00	9.00	6.69e-10
	Mutect2	0.470	1.13e-03	−9.00	9.00	3.92e-07
pfdhfr	HaplotypeCaller	0.863	6.87e-09	−15.37	16.91	2.60e-04
	Mutect2	0.771	2.54e-06	−9.20	13.30	1.45e-03
pfdhps	HaplotypeCaller	0.875	2.40e-09	−9.00	12.73	1.27e-02
	Mutect2	0.790	9.61e-05	−9.55	14.15	4.50e-02
pfmrp1	HaplotypeCaller	0.624	5.01e-04	+0.65	9.00	7.64e-01
	Mutect2	0.719	2.38e-05	+3.10	9.00	5.40e-01
pfmrp2	HaplotypeCaller	0.771	2.28e-15	−9.00	15.05	1.96e-03
	Mutect2	0.377	1.09e-03	−9.65	17.00	2.55e-04

Spearman ρ = Spearman rank correlation coefficient between expected and observed variant allele frequencies (VAFs). Spearman p-value = significance of correlation. Global bias (pp) = median difference in percentage points (observed – expected VAF). Global MAE (pp) = mean absolute error in percentage points. Global p_Wilcoxon = p-value from paired Wilcoxon signed-rank test across all dilutions. Negative bias values indicate systematic underestimation of the resistant allele frequency relative to expectation.

Table S7. Mixture quantification metrics by dilution ratio for Mutect2 and HaplotypeCaller, related to Figure 3.

Gene	Caller	Ratio (3D7:Dd2)	Bias (pp)	MAE (pp)	Wilcoxon p
pfcrt	Mutect2	1:1	-14,1	14,1	0.012
		2:1	-14,1	14,1	0.062
		5:1	-5,2	5,3	0.770
		10:1	-3,45	3,45	0.014
	HaplotypeCaller	1:1	-13,31	13,31	2.54×10^{-5}
		2:1	-15,96	15,96	0.007
		5:1	-5,84	5,84	0.001
		10:1	-9	9	0.0001
pfdhfr	Mutect2	1:1	-18,7	20	0.129
		2:1	-8,9	8,9	0.250
		5:1	-13,3	13,3	0.008
		10:1	-4,95	4,95	0.035
	HaplotypeCaller	1:1	-19,32	21,05	0.097
		2:1	-10,23	10,23	0.250
		5:1	-17	17	0.006
		10:1	-9	9	0.019
pfdhps	Mutect2	1:1	-11,5	17,4	1.000
		2:1	1,55	1,55	1.000
		5:1	-16,54	16,54	0.034
		10:1	-7,99	7,99	0.125
	HaplotypeCaller	1:1	-12,73	15,4	0.910
		2:1	1,98	1,98	0.500
		5:1	-17	17	0.007
		10:1	-9	9	0.019
pfmrp1	Mutect2	1:1	15,7	16,7	0.041
		2:1	-2,6	2,6	0.083
		5:1	0,6	8	0.221
		10:1	1,15	8,75	0.318
	HaplotypeCaller	1:1	4,55	30	0.006
		2:1	-3,83	3,83	0.012
		5:1	-0,33	7,56	0.044
		10:1	-1,98	7,89	0.057
pfmrp2	Mutect2	1:1	-14,75	24,4	0.029
		2:1	-9,2	9,2	0.071
		5:1	-17	17	0.165
		10:1	-7,55	7,9	0.228
	HaplotypeCaller	1:1	10,37	20,17	0.009
		2:1	-9,92	9,92	0.016
		5:1	-17	17	0.038
		10:1	-9	9	0.064

Ratio (3D7:Dd2) denotes the input proportion of 3D7 and Dd2 used to generate the mock mixtures and define the expected VAF for each dilution. Bias (pp) represents the median difference in percentage points between observed and expected VAF (observed – expected). MAE (pp) corresponds to the median absolute deviation in percentage points from the expected VAF. Wilcoxon p indicates the p-value from a paired Wilcoxon signed-rank test evaluating the observed–expected VAF differences for each gene–caller–dilution combination. Negative Bias values reflect systematic underestimation of the Dd2 allele frequency, whereas positive values reflect overestimation.

Table S8. Quantitative performance metrics by gene, parasite density, and mixture ratio, related to Figure 3.

Caller	Gene	Parasitemia (p/µL)	1:1 (Bias/MAE)	2:1 (Bias/MAE)	5:1 (Bias/MAE)	10:1 (Bias/MAE)	Spearman ρ
**HaplotypeCaller**	*pfcrt*	10	-50.00/50.00	NA	-17.00/17.00	NA	NA
**HaplotypeCaller**	*pfcrt*	100	-7.04/7.04	NA	-3.56/3.56	-9.00/9.00	0.96*
**HaplotypeCaller**	*pfcrt*	1000	-10.26/10.26	-15.96/15.96	-3.15/4.62	-9.00/9.00	0.90*
**HaplotypeCaller**	*pfdhfr*	10	16.67/16.67	NA	-17.00/17.00	NA	0.93*
**HaplotypeCaller**	*pfdhfr*	100	-34.91/34.91	NA	-17.00/17.00	-9.00/9.00	0.84*
**HaplotypeCaller**	*pfdhfr*	1000	-19.32/19.32	-10.23/10.23	-7.51/7.51	-9.00/9.00	0.91*
**HaplotypeCaller**	*pfdhps*	10	41.61/41.61	NA	-17.00/17.00	NA	0.93*
**HaplotypeCaller**	*pfdhps*	100	-15.40/15.40	NA	-17.00/17.00	-9.00/9.00	0.84*
**HaplotypeCaller**	*pfdhps*	1000	-12.73/12.73	1.98/1.98	-3.84/3.84	-9.00/9.00	0.91*
**HaplotypeCaller**	*pfmrp1*	10	-50.00/50.00	NA	-17.00/17.00	NA	0.45
**HaplotypeCaller**	*pfmrp1*	100	30.00/30.00	NA	-0.33/0.65	-9.00/9.00	0.96*
**HaplotypeCaller**	*pfmrp1*	1000	1.52/3.01	-3.83/3.83	7.56/7.56	5.63/5.63	0.91*
**HaplotypeCaller**	*pfmrp2*	10	22.82/30.14	NA	-17.00/17.00	NA	0.80*
**HaplotypeCaller**	*pfmrp2*	100	18.01/18.01	NA	-17.00/17.00	-9.00/9.00	0.84*
**HaplotypeCaller**	*pfmrp2*	1000	-19.44/19.44	-9.92/9.92	-8.25/8.25	3.27/7.22	0.71*
**Mutect2**	*pfcrt*	10	-50.00/50.00	NA	-17.00/17.00	NA	NA
**Mutect2**	*pfcrt*	100	-9.10/9.10	NA	-2.40/3.10	-3.50/3.50	0.95*
**Mutect2**	*pfcrt*	1000	-12.30/12.30	-14.10/14.10	0.30/5.20	-3.40/3.40	0.91*
**Mutect2**	*pfdhfr*	10	17.70/17.70	NA	-17.00/17.00	NA	0.93*
**Mutect2**	*pfdhfr*	100	-34.20/34.20	NA	-13.30/13.30	-5.30/5.30	0.64
**Mutect2**	*pfdhfr*	1000	-18.70/18.70	-8.90/8.90	-7.40/7.40	-4.60/4.60	0.95*
**Mutect2**	*pfdhps*	10	41.45/41.45	NA	-17.00/17.00	NA	0.94
**Mutect2**	*pfdhps*	100	-17.40/17.40	NA	-15.74/15.74	-7.99/7.99	0.84*
**Mutect2**	*pfdhps*	1000	-11.50/11.50	1.55/1.55	-11.10/11.10	-5.45/5.45	0.91*
**Mutect2**	*pfmrp1*	10	16.70/40.00	NA	-17.00/17.00	NA	0.69
**Mutect2**	*pfmrp1*	100	28.60/28.60	NA	0.60/5.90	-9.00/9.00	0.95*
**Mutect2**	*pfmrp1*	1000	3.10/3.10	-2.60/2.60	6.60/6.60	8.50/8.50	0.89*
**Mutect2**	*pfmrp2*	10	20.40/29.50	NA	-17.00/17.00	NA	0.69*
**Mutect2**	*pfmrp2*	100	19.35/22.90	NA	-15.85/15.85	-8.65/8.65	0.38
**Mutect2**	*pfmrp2*	1000	-20.90/20.90	-9.20/9.20	-9.10/9.10	3.50/6.25	0.27

Bias and MAE are expressed in percentage points (pp) and represent the median deviation between observed and expected variant allele frequencies and the median absolute error, respectively. Values are shown by gene, variant caller, parasite density, and mixture ratio (3D7:Dd2). Spearman's rho (ρ) reflects the monotonic agreement between observed and expected VAFs across mixture ratios for each gene and parasitemia level. Asterisks (*) indicate statistically significant correlations ($p < 0.05$). NA indicates unavailable estimates.

Table S9. Within-host heterozygosity across Mali, São Tomé and Príncipe and Brazil, related to subclonal variation in the Results section.

Population	n	Heterozygosity, median (IQR)	Heterozygosity, mean (sd)
**Mali**	44	0.0292 (0.0241–0.0335)	0.0286 (0.0156)
**São Tomé and Príncipe**	45	0.0194 (0.0152–0.0258)	0.0217 (0.0078)
**Brazil**	95	0.0158 (0.0119–0.0204)	0.0163 (0.0063)

Within-host heterozygosity was estimated as the proportion of heterozygous loci per sample. Values are presented as the number of samples (n), median with interquartile range (IQR), mean, and standard deviation (sd). Heterozygosity was calculated as the ratio of heterozygous genotype calls to the total number of successfully called loci per sample.

Table S10. Summary of duplex *pfcytb* qPCR assay conditions related to the STAR Methods section.

Category	Description
**Assay format**	Duplex hydrolysis probe qPCR targeting *P. falciparum pfcytb* and human *htubb*
***pfcytb* primers**	F: 5′-TGC AATT GTT AAA AAC TGT TCC AAG TA-3′ R: 5′-AGT TGT TAA ACT TCT TTG TTC TGC T-3′
***pfcytb* probe**	5′-(FAM)-CCA GCT GGT TTA GTA ATT GTA TTA TTA TCA-(BHQ1)-3′
***htubb* primers**	F: 5′-AAG GAG GTC GAT GAG CAG AT-3′ R: 5′-GCT GTC TTG ACA TTG TTG GG-3′
***htubb* probe**	5′-(Cy5)-TTA ACG TGC AGA ACA AGA ACA GCA GCT-(BHQ2)-3′
**Reaction conditions**	25 µL final volume containing 5 µL genomic DNA, 1× NH_4 buffer, 5.5 mM $MgCl_2$, 0.3 mM dNTPs, 0.3 µM primers, 0.2 µM probes, and 1 U Taq polymerase
**Cycling conditions**	95 °C for 3 min, followed by 40 cycles of 95 °C for 15 s, 56 °C for 15 s, and 72 °C for 30 s
**Quantification approach**	ΔCt normalization against *htubb* and ΔΔCt-based estimation relative to the WHO International Standard for *P. falciparum* DNA (49,000 parasites mL^{-1})
