# Supplementary material for: MalDeepSeq panel: A targeted ultra-deep sequencing approach to trace drug resistance markers in Plasmodium falciparum
Source: Cell Rep Methods. 2026 Jun 25;6(9):101509. doi: 10.1016/j.crmeth.2026.101509 (PMC13615499; doi:10.1016/j.crmeth.2026.101509)
Supplement: Supplementary file 2 [file mmc2.pdf]

Cell Reports
Methods

Article

MalDeepSeq panel: A targeted ultra-deep sequencing approach to trace drug resistance markers in *Plasmodium falciparum*

Graphical abstract

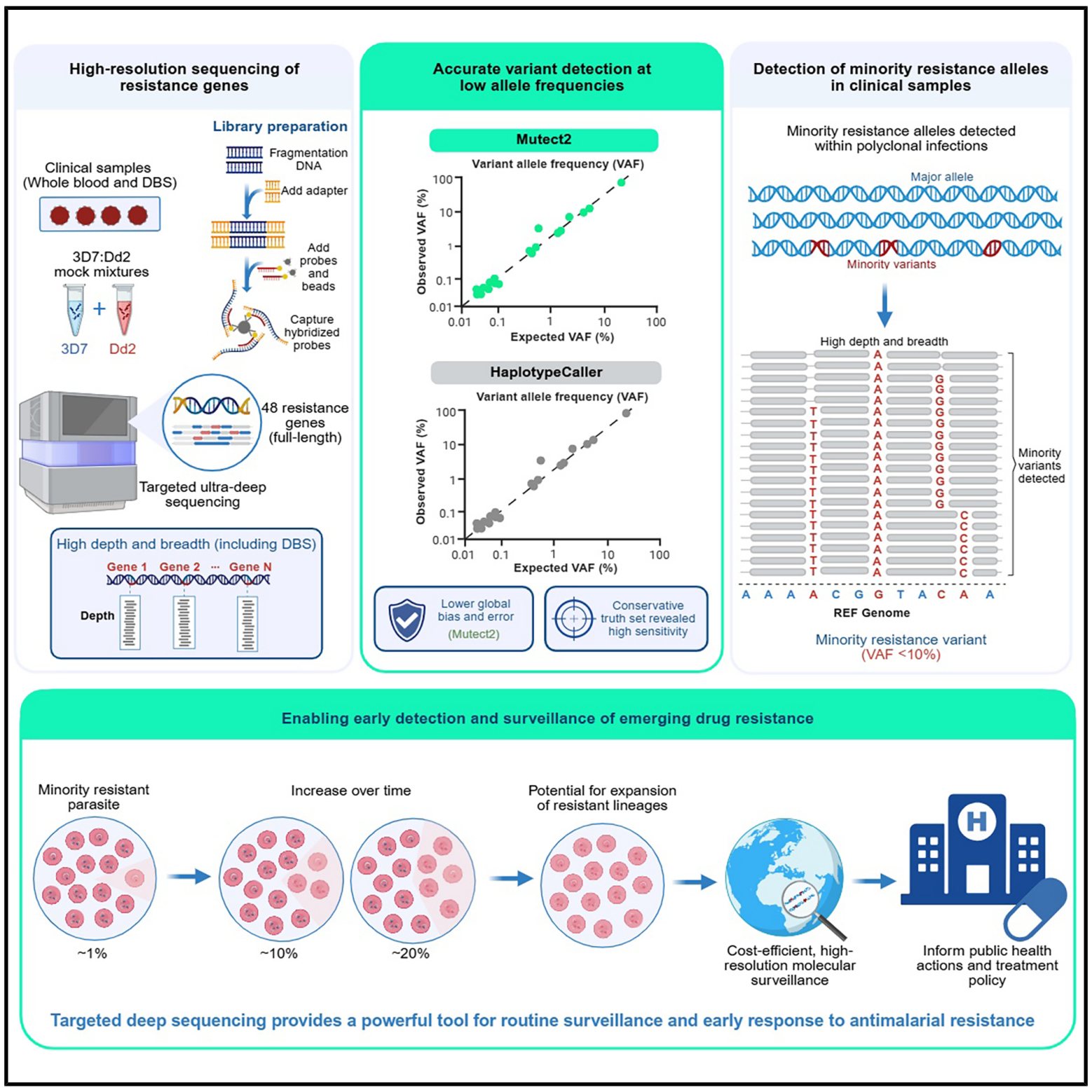

Highlights

- Targeted deep sequencing covers coding regions of 48 resistance-associated genes
- Detects minority variants in mixed *P. falciparum* infections
- Supports sequencing from whole blood and dried blood spots
- Enables scalable genomic surveillance of resistance

Authors

Yanka E.A. R. Salazar, Antonio M. Rezende, Maria C.S. B. Puça, ..., Dinora Lopes, Tais N. de Sousa, José P. Gil

Correspondence

tais.nobrega.de.sousa@ki.se

In brief

Salazar et al. developed a targeted deep sequencing approach for monitoring antimalarial resistance in *Plasmodium falciparum*. By combining broad genomic coverage with compatibility for dried blood spots, the method enables scalable detection of both dominant and minority resistant parasite populations in endemic settings.

Salazar et al., 2026, Cell Reports Methods *6*, 101509
September 21, 2026 © 2026 The Author(s). Published by Elsevier Inc.
https://doi.org/10.1016/j.crmeth.2026.101509

CellPress

Cell Reports Methods

CellPress
OPEN ACCESS

Article

MalDeepSeq panel: A targeted ultra-deep sequencing approach to trace drug resistance markers in *Plasmodium falciparum*

Yanka E.A. R. Salazar,[1,2,12] Antonio M. Rezende,[3,12] Maria C.S. B. Puça,[1,2] Sonja Lagström,[4] Danielle Fletcher,[5] Julian Muwanguzi-Karugaba,[6] Colin J. Sutherland,[6] Khalid B. Beshir,[6] Maria E.P. Mascarenhas,[1] Jaime Louzada,[7] Joseli Oliveira-Ferreira,[8] Abdoulaye Djimde,[9] Dinora Lopes,[10] Tais N. de Sousa,[1,2,11,13,*] and José P. Gil[2,10,11]

[1]Molecular Biology and Malaria Immunology Research Group, Instituto René Rachou, Fiocruz Minas, Fundação Oswaldo Cruz, Fiocruz, Belo Horizonte, Brazil
[2]Department of Microbiology, Tumor and Cell Biology, Karolinska Institutet, Solna, Sweden
[3]Biotechnology Applied to Pathogens Research Group, Instituto René Rachou, Fiocruz Minas, Fundação Oswaldo Cruz, Fiocruz, Belo Horizonte, Brazil
[4]Life Sciences and Diagnostics Markets Group, Agilent Technologies Sweden AB, Solna, Sweden
[5]Life Sciences and Diagnostics Markets Group, Agilent Technologies LDA UK Limited, Cheadle, UK
[6]Department of Infection Biology, Faculty of Infectious and Tropical Diseases, London School of Hygiene and Tropical Medicine, London, UK
[7]Universidade Federal de Roraima, Boa Vista, Brazil
[8]Laboratório de Imunoparasitologia, Instituto Oswaldo Cruz, Fundação Oswaldo Cruz, Fiocruz, Rio de Janeiro, Brazil
[9]Malaria Research and Training Center (MRTC), Faculty of Pharmacy, University of Science, Techniques and Technologies of Bamako, Bamako, Mali
[10]Clinical Tropical Medicine (CTM), Global Health and Tropical Medicine, Institute of Hygiene and Tropical Medicine, Nova University of Lisbon, Lisbon, Portugal
[11]Senior author
[12]These authors contributed equally
[13]Lead contact
*Correspondence: tais.nobrega.de.sousa@ki.se
https://doi.org/10.1016/j.crmeth.2026.101509

MOTIVATION Current approaches for *Plasmodium falciparum* resistance surveillance often rely on limited genomic targets or predefined hotspot mutations, restricting the detection of emerging variants and minority resistant subpopulations. In addition, whole-genome sequencing remains costly and operationally challenging for large-scale implementation in endemic settings. We developed a targeted deep sequencing panel covering the full coding regions of 48 resistance-associated genes to enable scalable, sensitive, and field-adapted genomic surveillance using both whole blood and dried blood spots.

SUMMARY

The emergence of drug-resistant *Plasmodium falciparum* highlights the need for tools to detect minor parasite subpopulations before resistant lineages expand. We developed and validated a targeted ultra-deep sequencing framework for the full-length sequences of 48 antimalarial resistance genes. Performance was evaluated using 3D7:Dd2 mock mixtures and clinical samples after selective whole-genome amplification. The panel achieved high depth and breadth, including for dried blood spot samples. Using Dd2-fixed markers, Mutect2 and HaplotypeCaller reproduced expected variant-allele frequencies, with Mutect2 exhibiting lower global bias and error. A conservative truth set derived from Dd2-pure controls yielded perfect recall and no false-positives in 3D7 controls, with high recovery of true Dd2 variants in mock mixtures for both callers. In clinical samples, the panel captured within-host diversity and minority resistance alleles. Overall, these results demonstrate that targeted deep sequencing offers a cost-efficient, high-resolution approach for routine molecular surveillance of emerging drug resistance.

© 2026 The Author(s). Published by Elsevier Inc.
This is an open access article under the CC BY license (http://creativecommons.org/licenses/by/4.0/).

INTRODUCTION

Drug-resistant *Plasmodium falciparum* lineages continue to emerge and spread, threatening decades of progress in malaria control.[1,2] In high-transmission settings, the complexity of polyclonal infections can obscure minority parasite lineages carrying resistance alleles, making the early emergence of resistant variants difficult to detect. The ability to capture such important minority parasite subpopulations carrying resistance alleles is a challenge in molecular surveillance. Those subpopulations often remain undetected by conventional methods but can expand rapidly under drug pressure.[3,4] Early and sensitive detection of variants assigning the resistant phenotype is therefore essential to guide treatment policies and safeguard frontline therapies.

Traditional molecular surveillance methods, such as single-gene PCR assays or Sanger sequencing, have provided important insights but remain limited in throughput, scalability and sensitivity, particularly when working with field-collected material such as dried blood spots (DBSs),[5] which often contain low parasite load and high levels of host DNA contamination. On the other side, while whole genome sequencing (WGS) provides comprehensive information, its high cost and computational demand (both infrastructure and human expertise) limit its widespread use in endemic settings.[6] In contrast, targeted next-generation sequencing (NGS) panels provide a scalable and cost-effective alternative by enabling multiplexed analyses of molecular markers.[7] Several studies have demonstrated the feasibility of this approach in malaria surveillance,[8–10] though their breadth and validation vary across sample types, parasite densities, and epidemiological contexts. Most existing targeted sequencing assays, however, have focused on a limited number of resistance genes or on hotspot regions within them,[8,10–13] whereas an approach which retains read-depth while providing complete coding coverage across key resistance-associated loci would offer expanded genomic resolution for surveillance.

Here, we describe a 48-gene targeted sequencing panel to track *P. falciparum* resistance-associated genes and performed its validation. The panel was evaluated using both controlled mock mono and multiclonal infections of *P. falciparum* strains 3D7 and Dd2, and clinical samples from different malaria settings, spanning whole blood (WB) and DBS with varying parasite densities. We assessed coverage, reproducibility, and quantitative performance, with emphasis on the detection of minority resistance alleles. Together, these analyses establish a versatile tool for molecular surveillance that is optimized for challenging sample types and capable of enhancing the early detection of drug-resistant variants that would likely be missed by conventional approaches.

RESULTS

Panel-wide performance across targets in mock infections

A graphical summary of the study workflow, highlighting the key steps from sample processing through variant analysis, is shown in Figure 1. Across the 48-gene panel, CDS depth was consistently high, with most loci exceeding hundreds to thousands of reads and only a few showing locus-specific variability (Figures 2A and 2B). CDS breadth of coverage mirrored these patterns, with nearly all genes achieving complete coverage across mock infections; reductions were confined to a small set of loci, mainly in low-yield material (blue points, Figure 2C). Together, these results demonstrate that the panel provides robust amplification and sequencing efficiency across diverse genomic targets.

Performance by DNA input subset and depth threshold

In mock infections, assay performance scaled with parasite input. A locus was defined as successful when ≥80% of its coding sequence (CDS breadth) reached the specified depth threshold (10×, 30×, or 80×). At 1,000 parasites, median depth was 385× with 93.8%, 78.1%, and 51.0% of loci successful at 10×, 30×, and 80×, respectively. At 100 parasites, median depth was 231× with 81.2%, 62.5%, and 39.6% success; and at the detection limit of 10 parasites, median depth was 49× with 52.1%, 35.4%, and 22.9% success. As expected, the proportion of successful loci decreased with lower inputs and more stringent thresholds, reaching only 22.9% at 80× in the 10-parasite group.

Using per-base depths, we modeled the probability of achieving ≥80% CDS breadth across mock inputs (pure controls excluded from fitting). The estimated LoD95 was 9.05×10^2 parasites at 10× (95% CI 4.48×10^2–2.61×10^3), 3.29×10^4 at 30× (95% CI 8.75×10^3–3.22×10^5), and 1.18×10^7 at 80× (95% CI 5.12×10^5–1.48×10^{10}), confirming that higher depth thresholds demand much higher input. Because the logistic model extrapolates the probability curve beyond the tested inputs (10–1,000 parasites), LoD95 values exceed the experimental range, reflecting the input required to achieve 95% success under each threshold. In practice, 10× depth preserves sensitivity at low inputs, whereas 30×/80× are suitable mainly for higher-yield material.

Performance across DNA yield categories is summarized in Tables S3 and S4. The WB group achieved a median per-base depth of 698× across all loci, with 100% of loci meeting the breadth criterion (≥80% CDS) at 10×, 91.7% at 30×, and 72.9% at 80×. The DBS group showed comparatively lower coverage (550× median depth) and breadth (97.9% at 10×; 85.4% at 30×; 58.3% at 80×). Interestingly, the DBS-opt group performed better, achieving the highest median depth among the three subsets (860×) and the best breadth at stricter thresholds (100% at 10×; 95.8% at 30×; 85.4% at 80×). This likely reflects the combined effect of selective whole-genome amplification (SWGA) enrichment, the adapted pooling strategy, and differences in sample composition (e.g., lower background human DNA or reduced degradation in DBS), which together enhanced capture efficiency despite the initially limited amount of parasite DNA.

Depth profiles for key resistance genes in mock and clinical samples

Depth distributions were further examined for five key resistance genes: *pfcrt*, *pfmdr1*, *pfk13*, *pfdhfr*, and *pfdhps*. In mock infections, depth scaled predictably with parasite input, showing the widest variability at 10 parasites, especially for *pfcrt*, and stabilizing at 100 and 1,000 parasites (Figure 3A). Clinical samples followed similar patterns: the WB group achieved uniformly high

CellPress
OPEN ACCESS

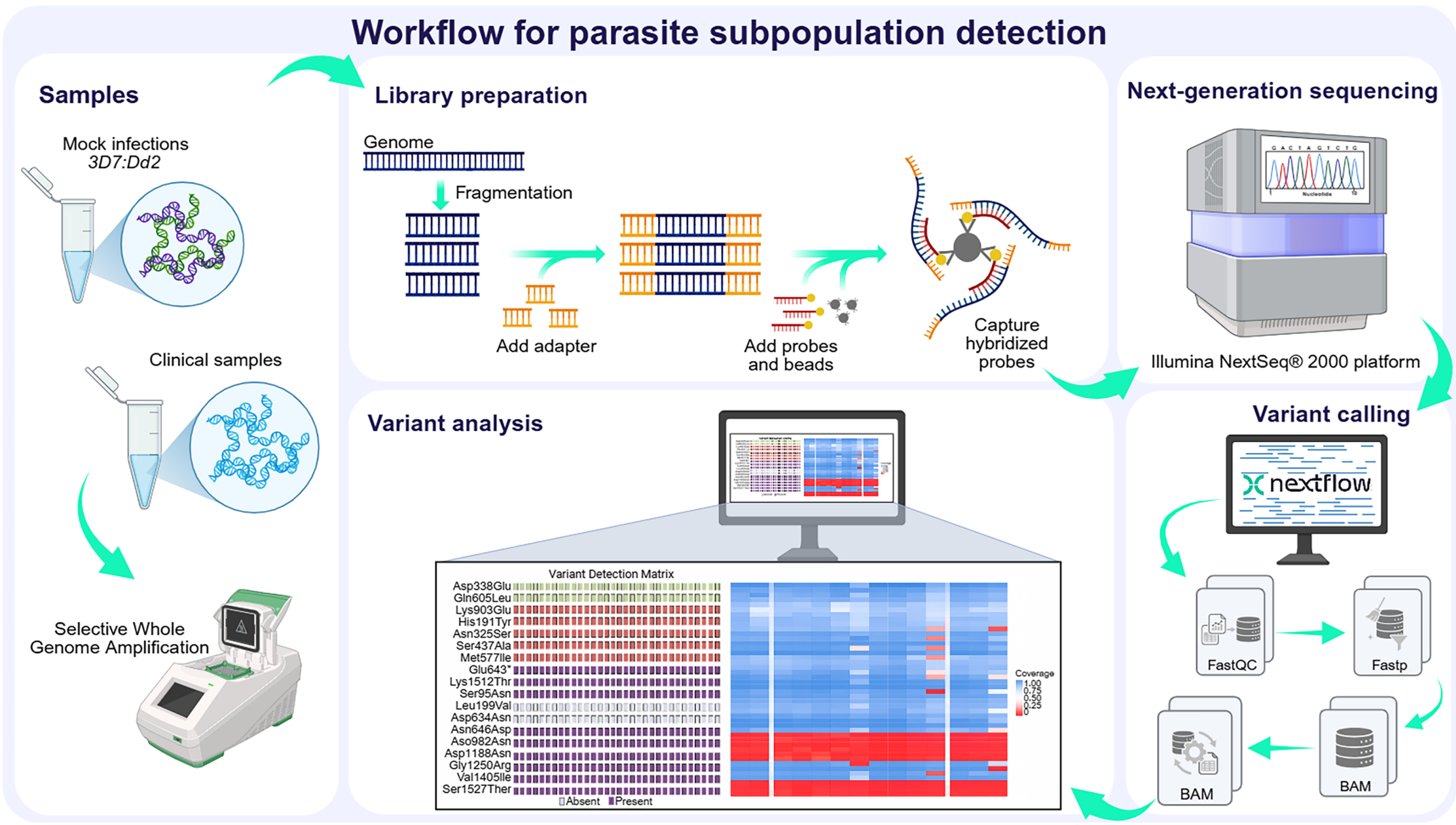

Figure 1. Workflow for parasite subpopulation detection using the MalDeepSeq panel

Overview of the targeted NGS workflow for *Plasmodium falciparum* variant detection. Mock infections (3D7:Dd2) and clinical samples were amplified by SWGA, followed by library preparation and target enrichment on the Agilent Magnis NGS Prep System and sequenced on the Illumina NextSeq 2000. Data were processed through an automated Nextflow pipeline for quality control, alignment, and variant calling. Downstream analyses included variant annotation and identification of minority parasite subpopulations. Created with BioRender.

depth across genes, while DBS and DBS-opt groups displayed greater variability. Nonetheless, the adapted DBS-opt workflow produced higher central depth values than the DBS group for most genes, with the exception of *pfk13* and *pfmdr1* (Figure 3A).

These findings indicate that, in our dataset, the main limitation at the DBS-opt group was coverage breadth: once loci achieved ≥80% CDS breadth at a given depth cutoff, the bases that were covered already reached depths well above the threshold. Thus, reliable recovery depended primarily on breadth, while depth at covered sites was generally sufficient for confident variant calling. To complement these results, base-by-base coverage profiles and read depth for the five genes are shown in Figure S1, demonstrating overall uniformity across CDS and highlighting localized drops in depth that may reflect sequence composition, GC-rich regions, or probe-capture performance.

Depth scales predictably with parasite density in key resistance genes

Median depth increased proportionally with parasite density across the same five resistance genes (Figure 3B). Variability was greatest at 10 parasites but diminished at higher inputs, underscoring the panel's ability to provide quantitative depth scaling across a wide input range. At the lowest input, the main limitation was incomplete gene coverage (Figure 2C), since sequenced regions already achieved hundreds of reads. All available replicates at 10, 100, and 1,000 parasites were pooled regardless of strain composition, providing a robust estimate of assay sensitivity under controlled conditions. This pattern was consistent with the results from clinical samples, where depth distributions also reflected the amount and quality of DNA yield (Figures S2–S4; Table S5). The DBS-opt group exhibited higher variability across loci but still achieved consistently high depth and coverage, likely reflecting intrinsic differences in capture efficiency rather than technical failure. The adapted workflow for this set of samples likely contributed to maintaining performance despite limited DNA quantities. Overall, these findings demonstrate the robustness of the panel across variable DNA yields, with locus-specific variation representing expected biological and design-related features rather than limitations of the assay.

Mixture quantification at resistance loci

To evaluate the panel's quantitative performance, we compared observed variant allele frequencies (VAFs) against theoretical expectations from 3D7:Dd2 mixtures. Dd2-fixed markers, defined as alleles present at ≥90% frequency in Dd2-pure controls, were used as reference sites (Figure 4). The curated truth set contained 21 Dd2-specific loci, which were used to benchmark caller performance across genes.

At *pfcrt*, both callers reproduced the expected dilution gradient, with moderate Spearman correlations (HaplotypeCaller: $\rho = 0.58$,

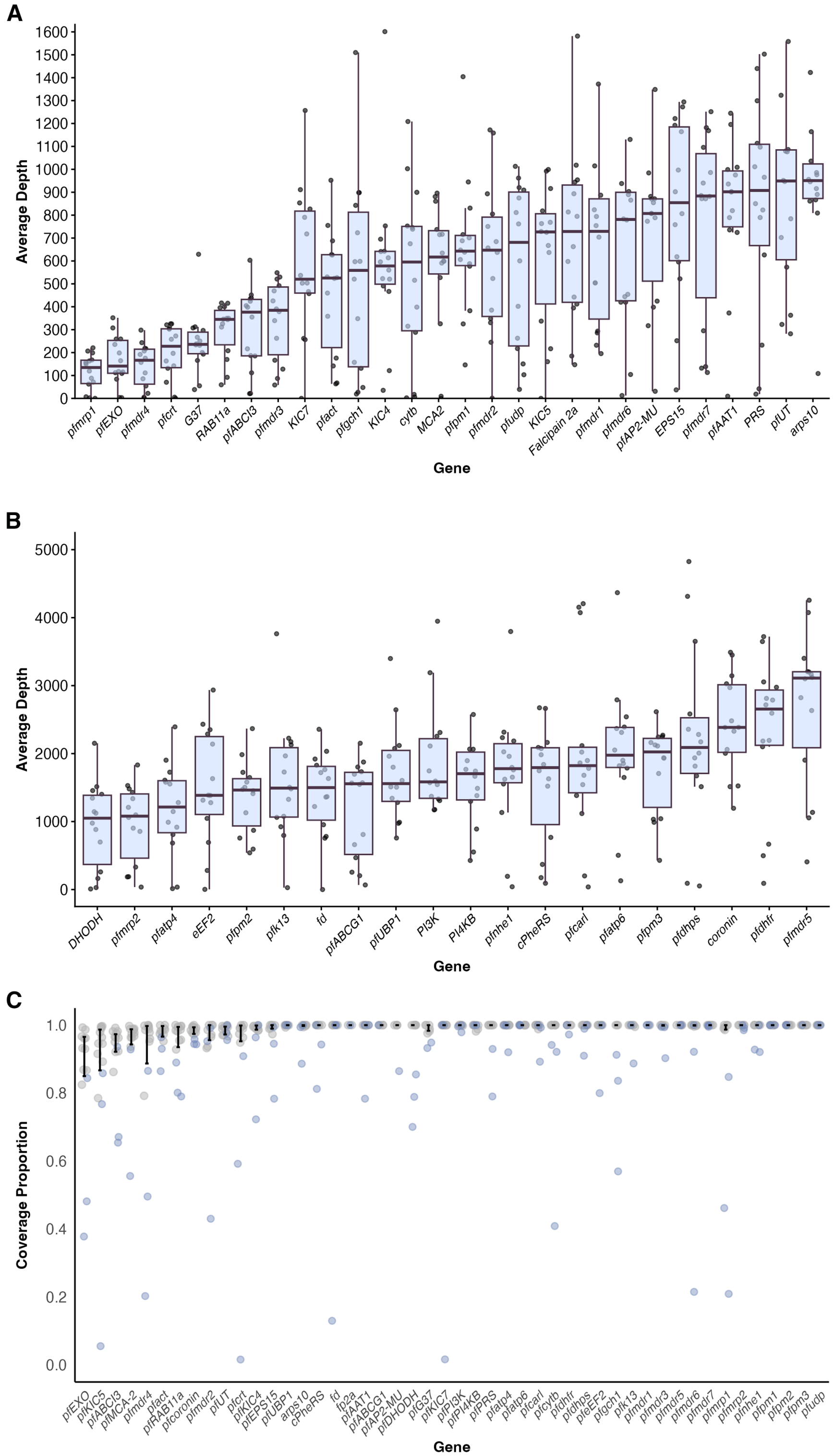

Figure 2. Sequencing depth and coverage breadth for mock infections across the 48-gene targeted panel

(A and B) Distribution of average read depth per gene across all samples, separated by whether the median depth was below (A) or above (B) 1,000×. Boxes represent interquartile ranges (IQRs), whiskers indicate data spread, and points show individual samples. The split highlights loci with lower versus consistently high sequencing performance.

(C) Base-level coverage breadth per gene (points), with black error bars denoting the IQR. Blue points correspond to 10-parasite mock dilutions, illustrating increased variability under limiting input while preserving high breadth for most loci. Mock infections were generated by combining *P. falciparum* 3D7 and Dd2 strains at defined ratios (1:1, 2:1, 5:1, and 10:1) and parasite densities (10, 100, and 1,000 parasites), providing controlled mixtures to benchmark panel performance under different DNA input conditions.

$p = 3.7 \times 10^{-6}$; Mutect2: $\rho = 0.47$, $p = 1.1 \times 10^{-3}$). Global accuracy was similar for the two callers (bias = −9.0 pp; mean absolute error [MAE] = 9.0 pp) (Table S6). However, at the most diluted 10:1 mixture, Mutect2 showed noticeably smaller deviation (bias = −3.5 pp; MAE = 3.5 pp) compared with HaplotypeCaller (bias = −9.0 pp; MAE = 9.0 pp) (Table S7), indicating better stability in recovering low-frequency Dd2 signals.

Across loci, the expected VAF was determined by the mixture ratio, whereas measurement precision depended on total DNA input. Analysis of low-frequency variants (0%–20% VAF; Figure S5) revealed that dispersion decreases in more asymmetric mixtures (e.g., 1:10). Consistently, stratified analysis showed that dispersion is inversely correlated with DNA quantity, with higher variability observed at lower input levels (Table S8). Together, these results indicate that VAF estimates are influenced by both allelic proportion and total DNA input, with improved stability at higher DNA concentrations.

At *pfdhfr*, both callers showed strong monotonic agreement with expected ratios ($\rho = 0.86$ for HaplotypeCaller; 0.77 for Mutect2) (Table S6). Overall, Mutect2 produced VAF estimates closer to the expected values (bias = −9.2 pp; MAE = 13.3 pp) than HaplotypeCaller (bias = −15.4 pp; MAE = 16.9 pp). Both callers tended to underestimate the Dd2 fraction in balanced (1:1) mixtures, and this systematic deviation was similar between them; Mutect2 showed only a slightly smaller median deviation, consistent with its lower global bias.

In contrast, *pfdhps* was robustly detected by both callers, with high correlations (HaplotypeCaller: $\rho = 0.88$; Mutect2: $\rho = 0.79$)

CellPress
OPEN ACCESS

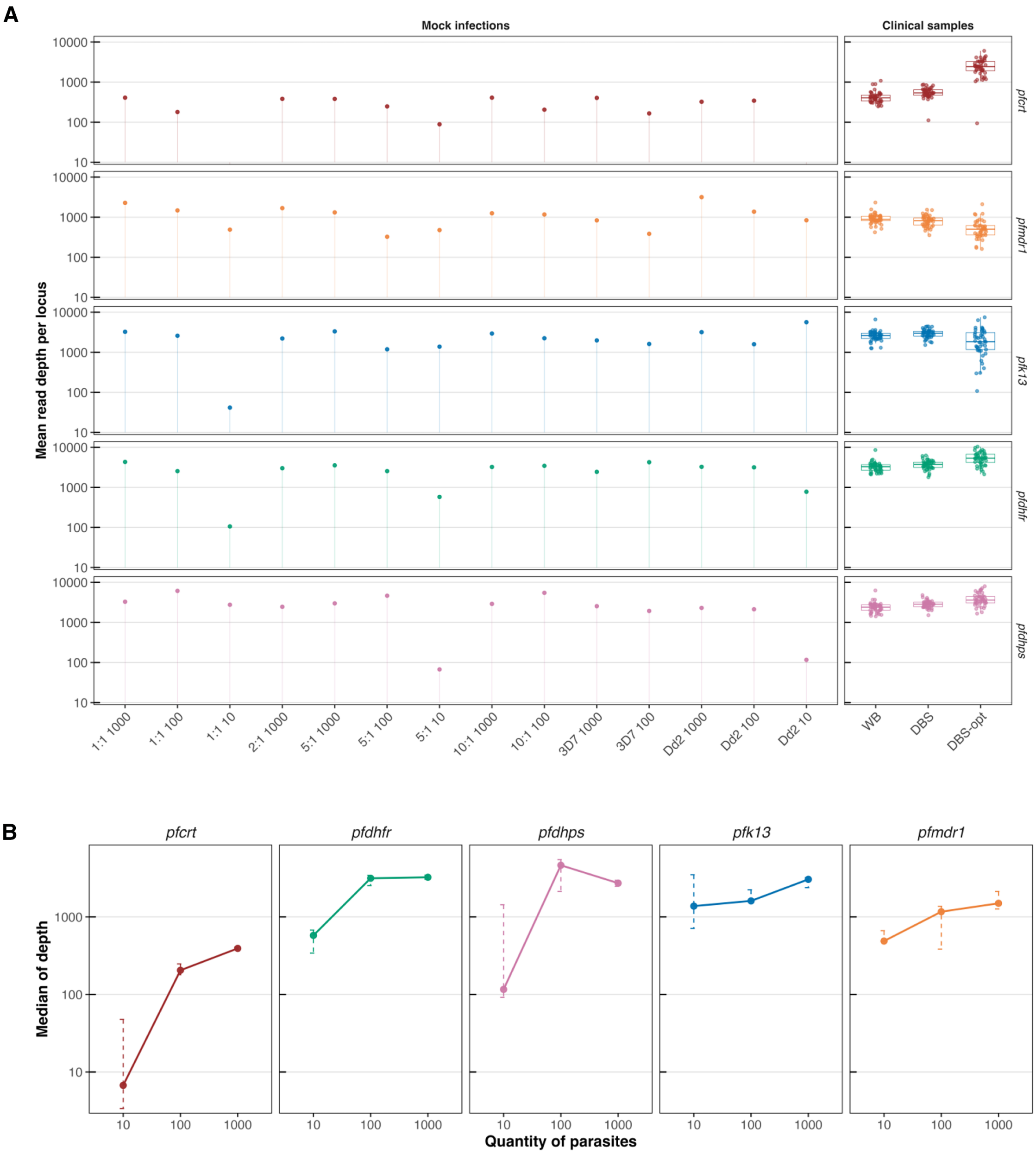

Figure 3. Depth performance across clinical and mock infections

(A) Read-pair depth distributions in clinical and mock infections. Mean read-pair per locus (log10 scale) is shown side-by-side for mock infections (left) and clinical subsets (right). In mock experiments (3D7:Dd2 ratio), depth scaled predictably with parasite density and was unaffected by strain composition. Among clinical samples, the whole-blood (WB) subset generated the tightest depth distributions, while the DBS-optimized (DBS-opt) group showed greater variability but higher central tendency than the DBS group, consistent with the beneficial impact of protocol adjustments. For clinical samples, boxes represent the interquartile range (IQR), the central black line indicates the median, and points represent individual samples. Mock infections are shown as individual observations.

(B) Depth behavior in mock dilutions across canonical resistance genes. Median depth (log10 scale) with IQRs for *pfcrt*, *pfdhfr*, *pfdhps*, *pfk13*, and *pfmdr1* at 10, 100, and 1,000 parasites. Depth scaled consistently with parasite density, with greater variability at 10 parasites and rapid stabilization thereafter. Medians were chosen to capture typical depth under variable low-yield conditions.

and comparable accuracy (global bias ≈ −9 pp; MAE ≈ 13–14 pp), with minimal qualitative differences.

Among additional loci, *pfmrp1* showed moderate agreement for both callers (ρ = 0.62 for HaplotypeCaller; 0.72 for Mutect2) and similar precision (global MAE = 9.0 pp). Mutect2 produced slightly higher VAF estimates (bias = +3.1 pp) than HaplotypeCaller (+0.7 pp).

pfmrp2 displayed the greatest dispersion overall, particularly for Mutect2 (ρ = 0.38; MAE = 17.0 pp). HaplotypeCaller performed somewhat better (ρ = 0.77; MAE = 15.1 pp), although

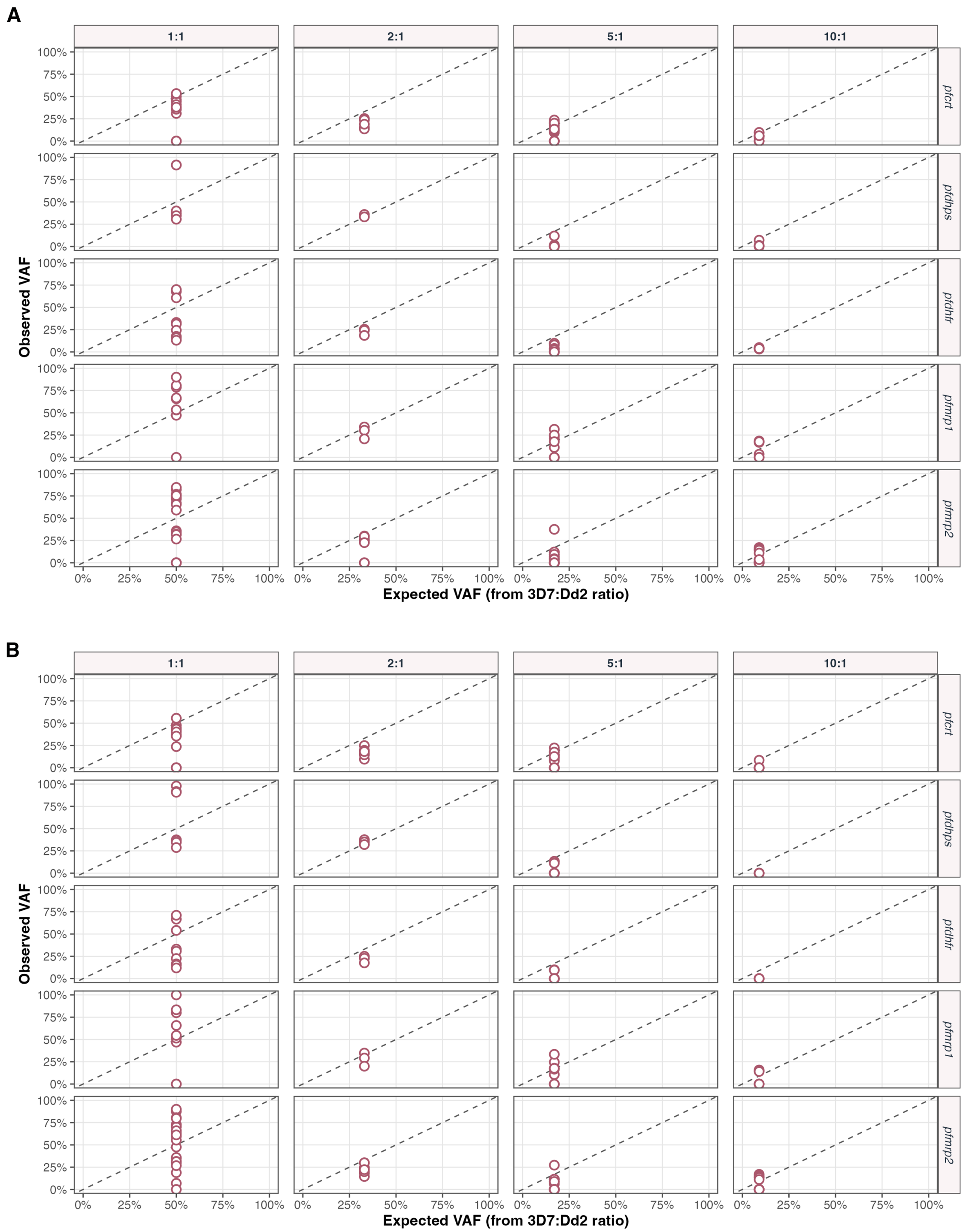

Figure 4. Quantification of 3D7:Dd2 mixtures at resistance loci using Mutect2 and HaplotypeCaller

(A and B) (A) Mutect2 and (B) HaplotypeCaller. Observed versus expected VAFs for *pfcrt, pfdhfr, pfdhps, pfmrp1* and *pfmrp2* across mock infections at 1:1, 2:1, 5:1, and 10:1 ratios. Each point represents the observed frequency of a Dd2-specific variant relative to 3D7 for the indicated gene. Dashed lines denote perfect concordance. Both callers produced VAF estimates for all 21 Dd2-specific loci in the truth set.

both callers systematically underestimated the Dd2 fraction (bias ≈ −9 pp). This reduced quantitative performance is consistent with known characteristics of *pfmrp2*,[14] which contains GC-rich regions, polymorphic flanking sequences, and lower capture efficiency in hybridization-based panels, all factors that can decrease enrichment uniformity and increase variance in allele-frequency estimates.

Overall, both callers correctly recapitulated the expected 3D7:Dd2 mixture gradient across the 21 Dd2-specific reference loci. Mutect2 delivered more quantitatively accurate VAF estimates at key resistance genes, particularly *pfcrt* and *pfdhfr*, and especially in low-proportion Dd2 mixtures, while HaplotypeCaller served as a complementary benchmark for variant detection accuracy (Tables S6 and S7).

To further characterize performance at the lowest experimentally tested minor-allele frequency, we performed a focused analysis of the 10:1 mixture (expected VAF ∼9%). At this dilution, both callers retained the ability to detect Dd2-specific alleles, but quantitative accuracy became increasingly gene-dependent. As described above, Mutect2 showed reduced deviation at *pfcrt* (bias = −3.5 pp; MAE = 3.5 pp) compared with HaplotypeCaller (bias = −9.0 pp; MAE = 9.0 pp), indicating improved stability in recovering low-frequency signals (Table S8). Similar patterns were observed for *pfdhfr*, where Mutect2 produced estimates closer to the expected values than HaplotypeCaller, whereas *pfdhps* showed comparable performance across callers. In contrast, *pfmrp2* displayed greater dispersion and reduced quantitative accuracy for both callers, consistent with locus-specific capture and sequence characteristics. Despite this variability, qualitative recovery of expected alleles remained high across all five benchmark genes, supporting robust detection of minor variants at approximately 9% frequency.

Variant detection performance against the truth set

To provide a conservative benchmark for qualitative detection accuracy, we constructed a truth set consisting of high-confidence Dd2-specific loci jointly supported by both callers in the Dd2-pure controls. These loci were required to show high representation in Dd2 (median VAF ≥ 90%) and no measurable background signal in 3D7-pure controls (median VAF ≤ 1%), resulting in a curated set of 103 validated Dd2 alleles. This consensus served as the reference for evaluating qualitative detection in the 3D7:Dd2 mixture experiments. No truth-set allele was detected in 3D7-pure samples by either caller, indicating the absence of false-positive events. Likewise, all truth-set loci were consistently detected in the Dd2-pure controls by both callers, demonstrating complete sensitivity in the pure strain background.

When restricting the analysis to the five resistance genes included in the mixture experiment (*pfcrt*, *pfdhfr*, *pfdhps*, *pfmrp1*, and *pfmrp2*), recovery of expected Dd2 alleles in the 3D7:Dd2 mixtures reached 100% for HaplotypeCaller (21/21 loci) and 95.2% for Mutect2 (20/21 loci). The single locus not recovered by Mutect2 corresponded to a *pfmrp2* allele that showed reduced coverage in mixed samples and consequently fell below Mutect2's internal quality filters, whereas it remained detectable by HaplotypeCaller. All remaining loci were successfully identified in at least one mixture sample by both callers.

These findings indicate that both variant callers exhibit high specificity and sensitivity in qualitative detection of validated Dd2 alleles. While Mutect2 applies stricter filtering, resulting in one missed locus, it provides more accurate quantitative VAF estimates across mixtures (as shown in the quantitative analysis), whereas HaplotypeCaller recovers the complete set of expected loci.

Subclonal variation across the 48-gene resistance panel

To ensure high confidence in minority-variant detection, particularly for low-frequency signals (VAF < 10%), we applied a conservative depth cutoff (DP ≥ 100×). Although this threshold is higher than the 80× criterion used for gene-level coverage metrics, it provides more stable VAF estimates and minimizes noise-driven false positives, an important requirement for interpreting subclonal variation. For each sample-gene pair, the maximum VAF was binned into discrete ranges (1%–10%, 10%–20%, 20%–30%, 30%–40%, 40%–50%, 50%–100%). Because a single high-VAF variant could mask the presence of multiple lower-frequency alleles, this summary was complemented by a majority-rule approach in which at least half of the evaluated sites within a gene had to fall within a given VAF band for that band to be assigned. This prevents isolated outlier variants from dominating the gene-level classification.

Across study populations, consistent low-frequency signals (1%–10% VAF) were observed in the major resistance genes (*pfk13*, *pfcrt*, *pfmdr1*, *pfdhfr*, and *pfdhps*) (Figure 5). Variants in the 10%–20% range were less common but still enriched in these loci, while many other genes were dominated by high-frequency alleles (50%–100%). All variants contributing to these summaries passed standard caller filters and met the DP ≥ 100× threshold, reducing the likelihood that low-frequency bands reflect strand imbalance, mapping artifacts, or other quality issues.

The enrichment of low-frequency variants within resistance genes suggests the presence of polyclonal infections and reflects distinct genetic patterns among the sampled populations. Such minority signals would likely remain undetected by conventional sequencing, underscoring the added value of high-resolution targeted sequencing to capture subclonal diversity in field isolates.

To provide additional insight into within-host genetic diversity, we assessed its variation across populations using the proportion of heterozygous loci as a proxy. This measure varied across populations, with higher values in high-transmission settings, consistent with differences in transmission intensity and infection complexity (Table S9).

DISCUSSION

This study presents the development and validation of a 48-gene targeted deep sequencing approach, designed to overcome key limitations in *P. falciparum* resistance surveillance. By combining broad genetic coverage with workflows optimized for both WB and DBS, the panel demonstrated robust performance across controlled mock infections and clinical specimens.

One of the main strengths of our approach is the design of a panel that covers the full length (∼293 kb) of 48 resistance-associated genes. Most previously published panels have focused on

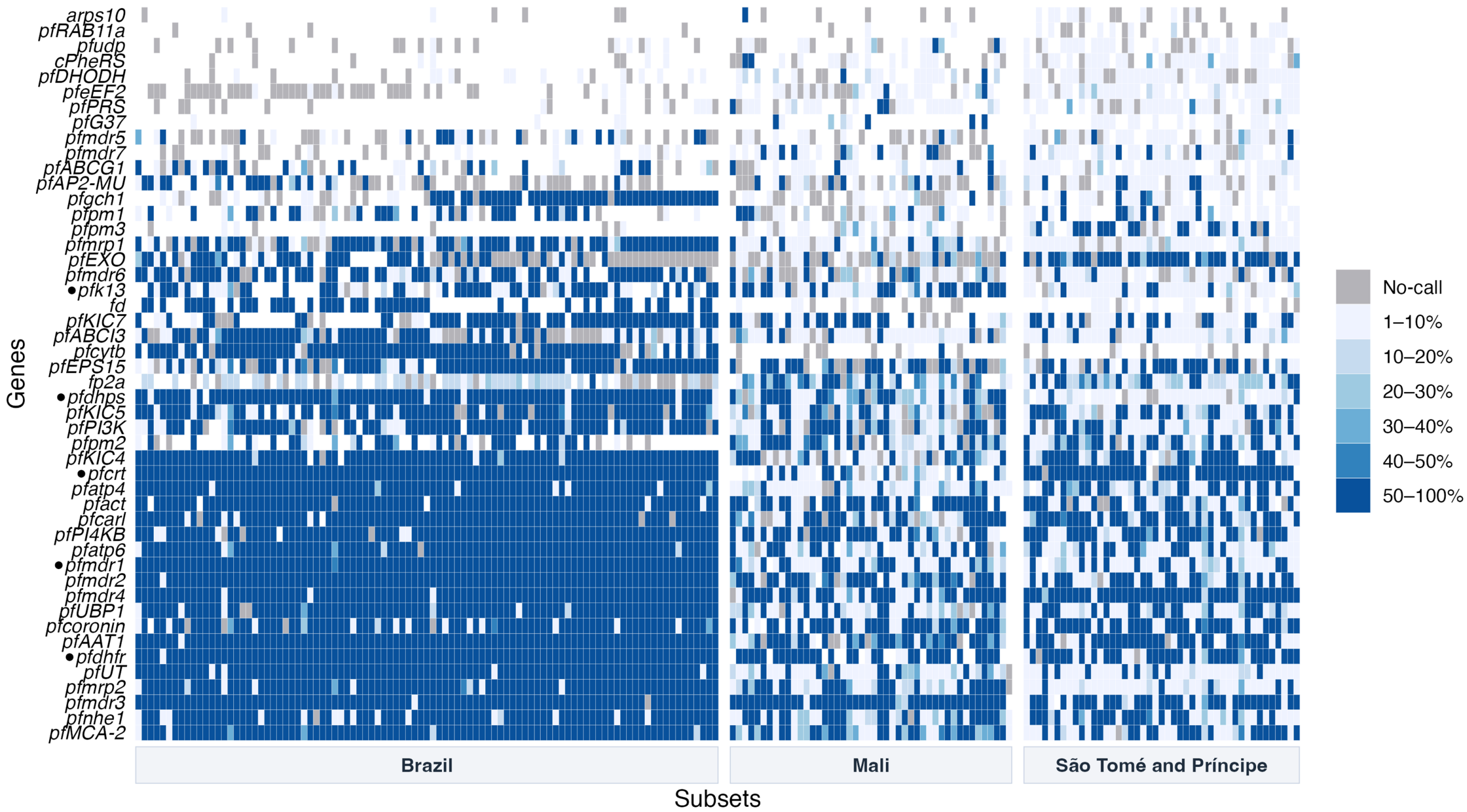

Figure 5. Subclonal allele frequency patterns across input cohorts

Heatmap of maximum VAFs per sample-gene pair, binned into six categories. Each column represents an individual sample, and each row a target gene within the 48-gene panel, with canonical resistance genes indicated by a bullet symbol (·). Gray = not evaluable (depth <100×); white = wild-type (no variant above threshold). Samples are grouped by population: Brazil, Mali, and São Tomé and Príncipe. Low-frequency alleles are consistently enriched at canonical resistance loci, indicating polyclonality across diverse parasite populations.

a smaller number of loci or restricted their design to known hotspot regions,[7,13] such as the MaRS protocol described previously,[7] which targets six key resistance genes using targeted amplicon deep sequencing, and more recent multiplexed approaches that prioritize predefined SNPs within selected loci.[13] More recently, another study developed the MAD4HatTeR panel,[15] a highly multiplexed amplicon sequencing approach focused on microhaplotypes, resistance markers, and population diversity, reinforcing the growing utility of targeted sequencing platforms for malaria genomic surveillance. While these studies demonstrated the feasibility of targeted sequencing for resistance surveillance, our panel provides a more comprehensive view of parasite genetic diversity by including entire coding regions. This extended coverage not only detects canonical markers but also enables the detection of novel variants and potential resistance mechanisms, an essential step for long-term surveillance in heterogeneous transmission settings.

Another distinctive feature of this work is the explicit evaluation of subclonal detection accuracy. The use of 10, 100, and 1,000 parasites/μL was designed to assess panel performance across low-to moderate-parasite densities used in validation studies of targeted amplicon sequencing panels, thereby enabling comparison with previous approaches. For instance, LaVerriere et al.[13] evaluated panel performance across similar parasite densities and demonstrated that sensitivity at low parasitaemia depends strongly on panel design and pre-amplification strategies. By using controlled 3D7:Dd2 mixtures, we were able to quantify bias and mean absolute error when comparing observed and expected VAFs. Both Mutect2 and HaplotypeCaller reproduced the expected trends, but Mutect2 consistently showed lower bias and smaller errors, yielding estimates closer to the expected values. This result is highly relevant for resistance surveillance, where minority parasite populations may indicate the early emergence of resistant lineages.

Notably, quantitative variability in balanced mixtures is not driven solely by allelic proportion. While mixture ratios define the expected VAF, the accuracy of its estimation depends on the amount of input DNA. Stratified analyses showed that dispersion increases at lower parasitemia levels, indicating that part of the observed variability arises from low-template effects rather than mixture proportion alone. In addition, the higher variability observed in balanced mixtures (1:1) suggests that cumulative technical biases play a more prominent role when alleles are present at similar proportions. This pattern is consistent with stochastic effects during SWGA and amplification-related biases, which are further compounded by sequencing-inherent factors associated with Illumina platforms, such as differential amplification efficiency and sequence-context effects, particularly under low-input conditions where uneven representation of haplotypes may occur.[16–18] Together, these findings indicate that VAF

CellPress
OPEN ACCESS

estimation reflects an interplay between allelic proportion and stochastic sampling, with balanced mixtures being more sensitive to technical fluctuations, whereas higher DNA input mitigates these effects by improving allele representation.

Previous studies using targeted amplicon sequencing in Africa and South America detected minority variants in clinical samples,[8,9,19] but our use of controlled 3D7:Dd2 mixtures provided a rigorous quantitative validation of caller performance, allowing direct measurement of bias and accuracy in minority allele detection.

Differences between callers can be attributed to their underlying models. While HaplotypeCaller, originally designed for germline variant detection, produced a higher total number of calls, this does not necessarily indicate superior performance. Mutect2, developed for detecting low-frequency variants in heterogeneous samples, applies probabilistic filters that prioritize accuracy over call quantity.[20] This explains its slightly lower number of calls but more consistent estimates of allele frequencies across mixtures. Our findings align with previous evaluations showing that Mutect2 achieves improved precision in mixed or low-allele-fraction samples compared to germline-oriented callers.[21]

The panel also proved adaptable to field-relevant materials. DBS are central to large-scale malaria surveillance but presents technical challenges due to low parasite DNA and human contamination.[5,22] By applying SWGA systematically and adapting purification steps for low-yield material, we ensured reliable coverage breadth in DBS from Mali and São Tomé and Príncipe. This demonstrates that with optimized protocols, DBS can yield quality data compatible with genomic monitoring of drug resistance.

Importantly, the panel was also validated using clinical samples collected across regions with moderate to high malaria endemicity, where a large proportion of infections are polyclonal.[23] In these settings, the panel accurately reflected the expected local molecular markers profiles and successfully detected minority subpopulations that would likely be missed by conventional genotyping or single-gene PCR approaches. This highlights its capacity to capture within-host diversity and to provide a more complete picture of parasite population structure in the field.

Scalability is another strength of our platform. With dual indexing, the assay allows multiplexing up to 192 libraries per run on the Illumina NextSeq 2000. Because only targeted regions are sequenced rather than the whole genome, high read depth is maintained even at this level of multiplexing. This ensures greater accuracy in allele frequency estimation, including for low-frequency variants. The analytical pipeline, implemented in Nextflow and publicly available, is lighter than whole-genome approaches, requiring less computational power and storage. These features facilitate adoption by laboratories with limited resources and support the integration of sequencing into national and regional malaria control programs.

While single-gene PCR assays remain cheaper on a per-target basis, performing them individually across all 48 genes would be substantially more expensive and less practical than using a single targeted sequencing assay. In contrast, our approach achieves broad locus coverage in one workflow, while still being considerably more cost-efficient than whole-genome sequencing, particularly when applied to large sample sets. Similar conclusions have been reported in other targeted approaches, where per-sample costs were reduced by high levels of multiplexing without compromising sensitivity.[11,12] By combining broad genetic coverage with efficient multiplexing, our panel achieves a balance between resolution, scalability, and affordability that makes targeted deep sequencing a practical option for surveillance in endemic regions where resources are constrained.

Therefore, our findings establish the 48-gene panel as a sensitive, versatile, and scalable tool for *P. falciparum* resistance monitoring. It reliably detects both dominant and minority variants, performs robustly with DBSs, and is supported by a standardized, publicly available workflow with moderate computational demands. While other targeted sequencing strategies have successfully demonstrated the feasibility of detecting minority variants and within-host diversity in field samples,[8,9,19] our approach extends this scope by combining CDS coverage of 48 resistance-associated genes with quantitative benchmarking in controlled mixtures and broad scalability. This unique combination of breadth, accuracy, and operational feasibility positions the panel as an innovative contribution to malaria molecular epidemiology and a valuable complement to existing regional and global surveillance strategies.[7,13,22]

This is critical in regions of significant infection complexity, typical of high transmission settings. Such complexity can largely obscure the early stages of drug resistance development, as minor resistant intra-infection subpopulations, still below the critical mass required to become clinically relevant, may be missed. These keep taking over the infection as drug pressure progressively selects them. Our approach allows the monitoring of this otherwise "invisible phase" of drug resistance development, permitting early warning of trends of resistance marker expansion.

This study has some inherent constraints typical of targeted deep sequencing applications. Although the panel performed consistently well across WB and DBS, targeted designs inherently sequence only predefined genomic regions and therefore cannot capture genome-wide variation or detect resistance mechanisms arising outside the selected loci. A further limitation is that the performance of mixture quantification was not evaluated at ratios below 10:1 ($\sim$9% minor-allele frequency). We therefore consider this threshold a conservative and experimentally supported limit for low-frequency detection at parasite densities $\geq$100 parasites/μL. Variants below this level are biologically relevant for resistance surveillance and may still be detectable depending on locus-specific depth and error profiles, but their accurate quantification was not directly validated here. Panel updates will also be necessary as new resistance-associated loci are identified. Its design allows relatively straightforward modification by adding or replacing probes targeting newly relevant genomic regions, thereby keeping it relevant. Finally, while this approach offers higher sensitivity and scalability than single-gene methods, it is not intended to replace WGS in

studies requiring full genomic context or structural variant discovery.

Limitations of the study

Although the panel showed high sensitivity for detecting low-frequency variants, this targeted approach is restricted to predefined coding regions and does not capture genome-wide diversity. Coverage may also vary across loci, particularly in samples with low parasite density or reduced DNA quality. In addition, the panel was specifically designed for *P. falciparum* and would require adaptation for use in other *Plasmodium* species or for broader genomic discovery applications.

RESOURCE AVAILABILITY

Lead contact

Requests for further information and resources should be directed to and will be fulfilled by the lead contact, Tais N. de Sousa (tais.nobrega.de.sousa@ki.se).

Materials availability

This study did not develop new materials or reagents.

Data and code availability

- Data generated and analyzed during this study are publicly available in the supplemental information and have been deposited in the NCBI Sequence Read Archive under BioProject accession numbers PRJNA1381748, PRJNA1470197, and PRJNA1353150.
- Custom scripts used for some of the analysis reported in this study are publicly available on Github and archived on Zenodo under permanent DOIs: https://github.com/yankaslzr/ (https://doi.org/10.5281/zenodo.20366906 and https://doi.org/10.5281/zenodo.20366905) and pipeline is available on https://github.com/AntonioRezende/malvariant/tree/master (https://doi.org/10.5281/zenodo.20384182).
- Any additional information required to re-analyze the data reported in this paper is available from the lead contact upon request.

ACKNOWLEDGMENTS

The authors thank all the patients who participated in this study. The authors are also grateful to the Karolinska Institutet Gene Core Facility, the National Genomics Infrastructure at SciLifeLab, and the Program for Technological Development in Tools for Health-PDTIS FIOCRUZ for the use of its facilities at René Rachou Institute. We sincerely thank Dr. Paulo Otavio Lourenço Moreira for creating the artwork used in Figure 1 and graphical abstract. M.C.S.B.P. thanks the René Rachou Institute for scholarship support. M.C.S.B.P., M.E.P.M., and Y.E.A.R.S. thank the Coordenação de Aperfeiçoamento de Pessoal de Nível Superior – Brasil (CAPES) for scholarship support (Finance Code 001) and the Graduate Program in Health Sciences at the René Rachou Institute. M.C.S.B.P. and Y.E.A.R.S. also thank the PrInt-Fiocruz-CAPES Program for scholarship support. We also thank the Federal University of Roraima (UFRR) for providing laboratory facilities and logistical support, which were essential for the development of this work. This work was supported by Fundação de Amparo à Pesquisa do Estado de Minas Gerais (FAPEMIG); the Swedish Research Council (Grant 200075/2022-5); the Conselho Nacional de Desenvolvimento Científico e Tecnológico (CNPq); WANECAM2, which is part of the EDCTP2 (RIA2017T-2018 WANECAM2); and the PrInt-Fiocruz-CAPES Program. T.N.S. and J.O.F. are recipients of CNPq Research Productivity Fellowships. Two authors (S.L. and D.F.) are employees of Agilent Technologies. The 4150 TapeStation System and the Magnis NGS Prep System used in this study were provided on loan by Agilent Technologies. The company and funders had no role in the study design, data collection, analysis, interpretation, or in the decision to submit the manuscript for publication.

AUTHOR CONTRIBUTIONS

Conceptualization, J.P.G. and T.N.S.; funding acquisition, J.P.G. and T.N.S.; methodology, Y.E.A.R.S., M.C.S.B.P., and M.E.P.M.; pipeline, A.M.R.; validation, Y.E.A.R.S. and A.M.R.; formal analysis, Y.E.A.R.S.; resources, J.L., J.O.F., S.L., D.F., J.M.K., C.S., K.B.B., A.D., and D.L.; data curation, Y.E.A.R.S. and T.N.S.; writing – original draft, Y.E.A.R.S. and T.N.S.; writing – review and editing, Y.E.A.R.S., A.M.R., J.P.G., and T.N.S. All authors read and approved the final manuscript.

DECLARATION OF INTERESTS

Two authors (S.L. and D.F.) are employees of Agilent Technologies. The 4150 TapeStation System and the Magnis NGS Prep System used in this study were provided on loan by Agilent Technologies. The company and funders had no role in the study design, data collection, analysis, interpretation, or in the decision to submit the manuscript for publication.

STAR★METHODS

Detailed methods are provided in the online version of this paper and include the following:

- KEY RESOURCES TABLE
- EXPERIMENTAL MODEL AND STUDY PARTICIPANT DETAILS
  - Sample sets, DNA extraction and parasite density quantitation
  - Parasite density quantification by qPCR
  - Validation of the duplex qPCR assay
- METHOD DETAILS
  - Panel design
  - Selective whole-genome amplification (SWGA)
  - Library preparation and sequencing
  - Bioinformatic processing
  - Variant annotation and coverage analysis
  - Limit-of-detection modeling in mock infections
  - Quantitative mixture analysis
  - Within-host diversity analysis
  - Truth set definition and qualitative evaluation
- QUANTIFICATION AND STATISTICAL ANALYSIS

SUPPLEMENTAL INFORMATION

Supplemental information can be found online at https://doi.org/10.1016/j.crmeth.2026.101509.

Received: December 16, 2025
Revised: April 12, 2026
Accepted: June 2, 2026
Published: June 25, 2026

REFERENCES

1. Menard, D., and Dondorp, A. (2017). Antimalarial drug resistance: a threat to malaria elimination. Cold Spring Harb. Perspect. Med. *7*, a025619. https://doi.org/10.1101/cshperspect.a025619.
2. Siddiqui, F.A., Liang, X., and Cui, L. (2021). Plasmodium falciparum resistance to ACTs: Emergence, mechanisms, and outlook. Int. J. Parasitol.: Drugs Drug Resist. *16*, 102–118. https://doi.org/10.1016/j.ijpddr.2021.05.007.
3. Amambua-Ngwa, A., Amenga-Etego, L., Kamau, E., Amato, R., Ghansah, A., Golassa, L., Randrianarivelojosia, M., Ishengoma, D., Apinjoh, T., Maïga-Ascofaré, O., et al. (2019). Major subpopulations of Plasmodium falciparum in sub-Saharan Africa. Science *365*, 813–816. https://doi.org/10.1126/science.aav5427.
4. Gosi, P., Lanteri, C.A., Tyner, S.D., Se, Y., Lon, C., Spring, M., Char, M., Sea, D., Sriwichai, S., Surasri, S., et al. (2013). Evaluation of parasite

subpopulations and genetic diversity of the msp1, msp2 and glurp genes during and following artesunate monotherapy treatment of Plasmodium falciparum malaria in Western Cambodia. Malar. J. *12*, 403. https://doi.org/10.1186/1475-2875-12-403.

5. Nain, M., Sinha, A., and Sharma, A. (2023). Dried blood spots: a robust tool for malaria surveillance in countries targeting elimination. J. Vector Borne Dis. *60*, 11–17.

6. Akoniyon, O.P., Adewumi, T.S., Maharaj, L., Oyegoke, O.O., Roux, A., Adeleke, M.A., Maharaj, R., and Okpeku, M. (2022). Whole Genome Sequencing Contributions and Challenges in Disease Reduction Focused on Malaria. Biology *11*, 587. https://doi.org/10.3390/biology11040587.

7. Talundzic, E., Ravishankar, S., Kelley, J., Patel, D., Plucinski, M., Schmedes, S., Ljolje, D., Clemons, B., Madison-Antenucci, S., Arguin, P.M., et al. (2018). Next-generation sequencing and bioinformatics protocol for malaria drug resistance marker surveillance. Antimicrob. Agents Chemother. *62*, e02474-17. https://doi.org/10.1128/AAC.02474-17.

8. Osoti, V., Akinyi, M., Wamae, K., Kimenyi, K.M., De Laurent, Z., Ndwiga, L., Gichuki, P., Okoyo, C., Kepha, S., Mwandawiro, C., et al. (2022). Targeted Amplicon Deep Sequencing for Monitoring Antimalarial Resistance Markers in Western Kenya. Antimicrob. Agents Chemother. *66*, e0194521. https://doi.org/10.1128/aac.01945-21.

9. L'Episcopia, M., Kelley, J., Djeunang Dongho, B.G., Patel, D., Schmedes, S., Ravishankar, S., Perrotti, E., Modiano, D., Lucchi, N.W., Russo, G., et al. (2021). Targeted deep amplicon sequencing of antimalarial resistance markers in Plasmodium falciparum isolates from Cameroon. Int. J. Infect. Dis. *107*, 234–241. https://doi.org/10.1016/j.ijid.2021.04.081.

10. Wamae, K., Ndwiga, L., Kharabora, O., Kimenyi, K., Osoti, V., de Laurent, Z., Wambua, J., Musyoki, J., Ngetsa, C., Kalume, P., et al. (2022). Targeted Amplicon deep sequencing of ama1 and mdr1 to track within-host P. falciparum diversity throughout treatment in a clinical drug trial. Wellcome Open Res. *7*, 95. https://doi.org/10.12688/wellcomeopenres.17736.1.

11. Kunasol, C., Dondorp, A.M., Batty, E.M., Nakhonsri, V., Sinjanakhom, P., Day, N.P.J., and Imwong, M. (2022). Comparative analysis of targeted next-generation sequencing for Plasmodium falciparum drug resistance markers. Sci. Rep. *12*, 5563. https://doi.org/10.1038/s41598-022-09474-5.

12. Tessema, S.K., Hathaway, N.J., Teyssier, N.B., Murphy, M., Chen, A., Aydemir, O., Duarte, E.M., Simone, W., Colborn, J., Saute, F., et al. (2022). Sensitive, Highly Multiplexed Sequencing of Microhaplotypes from the Plasmodium falciparum Heterozygome. J. Infect. Dis. *225*, 1227–1237. https://doi.org/10.1093/infdis/jiaa527.

13. LaVerriere, E., Schwabl, P., Carrasquilla, M., Taylor, A.R., Johnson, Z.M., Shieh, M., Panchal, R., Straub, T.J., Kuzma, R., Watson, S., et al. (2022). Design and implementation of multiplexed amplicon sequencing panels to serve genomic epidemiology of infectious disease: A malaria case study. Mol. Ecol. Resour. *22*, 2285–2303. https://doi.org/10.1111/1755-0998.13622.

14. Okombo, J., Abdi, A.I., Kiara, S.M., Mwai, L., Pole, L., Sutherland, C.J., Nzila, A., and Ochola-Oyier, L.I. (2013). Repeat polymorphisms in the low-complexity regions of Plasmodium falciparum ABC transporters and associations with in vitro antimalarial responses. Antimicrob. Agents Chemother. *57*, 6196–6204. https://doi.org/10.1128/AAC.01465-13.

15. Aranda-Díaz, A., Neubauer Vickers, E., Murie, K., Palmer, B., Hathaway, N., Gerlovina, I., Boene, S., García-Ulloa, M., Cisteró, P., Katairo, T., et al. (2025). Sensitive and modular amplicon sequencing of Plasmodium falciparum diversity and resistance for research and public health. Sci. Rep. *15*, 10737. https://doi.org/10.1038/s41598-025-94716-5.

16. Cowell, A.N., Loy, D.E., Sundararaman, S.A., Valdivia, H., Fisch, K., Lescano, A.G., Baldeviano, G.C., Durand, S., Gerbasi, V., Sutherland, C.J., et al. (2017). Selective whole-genome amplification is a robust method that enables scalable whole-genome sequencing of Plasmodium vivax from unprocessed clinical samples. mBio *8*, e02257-16. https://doi.org/10.1128/mBio.02257-16.

17. Depristo, M.A., Banks, E., Poplin, R., Garimella, K.V., Maguire, J.R., Hartl, C., Philippakis, A.A., Del Angel, G., Rivas, M.A., Hanna, M., et al. (2011). A framework for variation discovery and genotyping using next-generation DNA sequencing data. Nat. Genet. *43*, 491–498. https://doi.org/10.1038/ng.806.

18. Aird, D., Ross, M.G., Chen, W.S., Danielsson, M., Fennell, T., Russ, C., Jaffe, D.B., Nusbaum, C., and Gnirke, A. (2011). Analyzing and minimizing PCR amplification bias in Illumina sequencing libraries. Genome Biol. *12*, R18. https://doi.org/10.1186/gb-2011-12-2-r18.

19. Aydemir, O., Mensah, B., Marsh, P.W., Abuaku, B., Myers-Hansen, J.L., Bailey, J.A., and Ghansah, A. (2021). Immediate pools of malaria infections at diagnosis combined with targeted deep sequencing accurately quantifies frequency of drug resistance mutations. PeerJ *9*, e11794. https://doi.org/10.7717/peerj.11794.

20. Broad Institute (2025). GATK Best Practices Workflows (Cambridge, MA: Broad Institute). https://gatk.broadinstitute.org.

21. Wang, Q., Kotoula, V., Hsu, P.C., Papadopoulou, K., Ho, J.W.K., Fountzilas, G., and Giannoulatou, E. (2019). Comparison of somatic variant detection algorithms using Ion Torrent targeted deep sequencing data. BMC Med. Genomics *12*, 181. https://doi.org/10.1186/s12920-019-0636-y.

22. Ghansah, A., Kamau, E., Amambua-Ngwa, A., Ishengoma, D.S., Maiga-Ascofare, O., Amenga-Etego, L., Deme, A., Yavo, W., Randrianarivelojosia, M., et al.; Plasmodium Diversity Network Africa (2019). Targeted Next Generation Sequencing for malaria research in Africa: Current status and outlook. Malar. J. *18*, 324. https://doi.org/10.1186/s12936-019-2944-2.

23. Lopez, L., and Koepfli, C. (2021). Systematic review of Plasmodium falciparum and Plasmodium vivax polyclonal infections: Impact of prevalence, study population characteristics, and laboratory procedures. PLoS One *16*, e0249382. https://doi.org/10.1371/journal.pone.0249382.

24. Salazar, Y.E.A.R., Rezende, A.M., Puça, M.C.S.B., Louzada, J., Mascarenhas, M.E.P., Lagström, S., Fletcher, D., Oliveira-Ferreira, J., Gil, J.P., and de Sousa, T.N. (2026). Emergence of artemisinin-based combination therapy resistance markers in Plasmodium falciparum from the Brazilian triborder region of the Guiana Shield. Antimicrob. Agents Chemother. *70*, e0152525. https://doi.org/10.1128/aac.01525-25.

25. Gardner, M.J., Hall, N., Fung, E., White, O., Berriman, M., Hyman, R.W., Carlton, J.M., Pain, A., Nelson, K.E., Bowman, S., et al. (2002). Genome sequence of the human malaria parasite Plasmodium falciparum. Nature *419*, 498–511. https://doi.org/10.1038/nature01097.

26. Luth, M.R., Godinez-Macias, K.P., Chen, D., Okombo, J., Thathy, V., Cheng, X., Daggupati, S., Davies, H., Dhingra, S.K., Economy, J.M., et al. (2024). Systematic in vitro evolution in Plasmodium falciparum reveals key determinants of drug resistance. Science *386*, eadk9893. https://doi.org/10.1126/science.adk9893.

27. Robinson, A., Busula, A.O., Muwanguzi, J.K., Powers, S.J., Masiga, D.K., Bousema, T., Takken, W., de Boer, J.G., Logan, J.G., Beshir, K.B., and Sutherland, C.J. (2019). Molecular quantification of Plasmodium parasite density from the blood retained in used RDTs. Sci. Rep. *9*, 5107. https://doi.org/10.1038/s41598-019-41438-0.

28. Beshir, K.B., Hallett, R.L., Eziefula, A.C., Bailey, R., Watson, J., Wright, S. G., Chiodini, P.L., Polley, S.D., and Sutherland, C.J. (2010). Measuring the efficacy of anti-malarial drugs in vivo: Quantitative PCR measurement of parasite clearance. Malar. J. *9*, 312. https://doi.org/10.1186/1475-2875-9-312.

29. Sawa, P., Shekalaghe, S.A., Drakeley, C.J., Sutherland, C.J., Mweresa, C. K., Baidjoe, A.Y., Manjurano, A., Kavishe, R.A., Beshir, K.B., Yussuf, R.U., et al. (2013). Malaria transmission after artemether-lumefantrine and dihydroartemisinin- piperaquine: A randomized trial. J. Infect. Dis. *207*, 1637–1645. https://doi.org/10.1093/infdis/jit077.

30. MalariaGEN Selective Whole Genome Amplification (sWGA): Protocol for amplification of Plasmodium falciparum. MalariaGEN. https://www.malariagen.net/resources/amplicon-sequencing-toolkit/p-falciparum-amplicontoolkit-protocols/
31. Van der Auwera, G.A., Carneiro, M.O., Hartl, C., Poplin, R., del Angel, G., Levy-Moonshine, A., Jordan, T., Shakir, K., Roazen, D., Thibault, J., et al. (2013). From fastQ data to high-confidence variant calls: The genome analysis toolkit best practices pipeline. Curr. Protoc. Bioinformatics *43*, 11.10.1–11.10.33. https://doi.org/10.1002/0471250953.bi1110s43.
32. Coonahan, E., Gage, H., Chen, D., Noormahomed, E.V., Buene, T.P., Mendes de Sousa, I., Akrami, K., Chambal, L., Schooley, R.T., Winzeler, E.A., and Cowell, A.N. (2023). Whole-genome surveillance identifies markers of Plasmodium falciparum drug resistance and novel genomic regions under selection in Mozambique. mBio *14*, e0176823. https://doi.org/10.1128/MBIO.01768-23.

CellPress
OPEN ACCESS

STAR★METHODS

KEY RESOURCES TABLE

REAGENT or RESOURCE	SOURCE	IDENTIFIER
**Biological samples**		
Whole-blood samples from Brazil	Salazar et al.[24]	N/A
Dried-blood spots samples from São Tomé and Príncipe	This paper	N/A
Dried-blood spots samples from Mali	This paper	N/A
**Chemicals, peptides, and recombinant proteins**		
Recombinant Albumin	New England Biolabs	Cat#B9200S
**Critical commercial assays**		
phi29 DNA Polymerase	New England Biolabs	Cat#M0269S
phi29 DNA Polymerase Reaction Buffer	New England Biolabs	Cat#B0269
SureSelect Custom Tier1	Agilent Technologies	Cat#3488401
Magnis SureSelect XT HS2 DNA, No Probe, ILMN, 96 Rxns	Agilent Technologies	Cat#G9750B
Genomic DNA Reagents	Agilent Technologies	Cat#5067-5366
High Sensitivity D1000 Reagents	Agilent Technologies	Cat#5067-5585
High Sensitivity D1000 Reagents	Agilent Technologies	Cat#5067-5585
AMPure XP magnetic beads	Backman Coulter	Cat#A63881
**Deposited data**		
Sequences		Bioproject: PRJNA1381748, PRJNA1470197, and PRJNA1353150
**Experimental models: organisms/strains**		
Plasmodium falciparum 3D7 strain	Karolinska Institutet laboratory	N/A
Plasmodium falciparum Dd2 strain	Karolinska Institutet laboratory	N/A
**Software and algorithms**		
FastQC v0.11.9	Babraham Bioinformatics	RRID: SCR_014583
MultiQC	https://github.com/MultiQC/MultiQC	RRID: SCR_014982
Picard Tools	Broad Institute	RRID: SCR_006525
BWA-MEM v0.7.18		RRID: SCR_022192
GATK4	Broad Institute	N/A
bcftools v1.20		RRID: SCR_005227
SnpEff v5.1		RRID: SCR_005191
MalDeepSeq Nextflow pipeline		https://github.com/AntonioRezende/malvariant/tree/master
Matplotlib		RRID: SCR_008624
Python	Python Software Foundation	RRID: SCR_00839
Seaborn v0.13.2	Waskom	RRID: SCR_018132
**Other**		
Genomic DNA ScreenTape	Agilent Technologies	Cat#5067-5365
High Sensitivity D1000 ScreenTape	Agilent Technologies	Cat#5067-5584
D1000 ScreenTape	Agilent Technologies	Cat#5067-5582

EXPERIMENTAL MODEL AND STUDY PARTICIPANT DETAILS

Sample sets, DNA extraction and parasite density quantitation

Performance of the panel was evaluated in both experimental mock infections and clinical specimens. Mock infections were generated using the *P. falciparum* laboratory strains 3D7 and Dd2. The 3D7 strain, originally isolated in Africa, is the reference genome

widely used in malaria research and represents a chloroquine-sensitive lineage.[25] In contrast, Dd2, derived from Southeast Asia, is a multidrug-resistant strain carrying well-characterized polymorphisms in resistance genes.[26] These two genetic backgrounds were selected to mimic multiclonal infections involving sensitive and resistant parasites, thereby providing a controlled system to test the panel's ability to detect minor variants.

Mock infections included pure and mixed ratios of 3D7:Dd2, which were diluted to 1000, 100, and 10 parasites by serial dilution in human DNA, following the protocol described in a previous study.[13] This design provided a controlled framework to assess the panel's sensitivity under limiting DNA input. Mock infections were prepared from DNA extracted using the QIAamp DNA Blood Mini Kit (Qiagen) from 200 μL of *P. falciparum* cultures (3D7 and Dd2 strains). DNA concentrations were quantified with a Qubit 4 fluorometer. To generate mock mono-infections of 10,000 parasites/μL, parasite DNA was mixed with human genomic DNA (Promega) to yield 0.25 ng/μL parasite DNA (0.25 ng corresponds to the mass of ∼10,000 haploid genomes, assuming a 23 Mbp genome and an average nucleotide mass of 660 $g.mol^{-1}.bp^{-1}$) in 10.0 ng/μL human DNA. For 3D7, parasite DNA at 1.1 ng/μL was combined with human DNA at 12.9 ng/μL in a 1:3.4 (v/v) parasite:human ratio to obtain the target concentrations. For Dd2, parasite DNA at 2.2 ng/μL was mixed with human DNA at 11.3 ng/μL in a 1:7.8 (v/v) ratio to achieve the same target concentrations. Mock mono-infections of 1,000, 100 and 10 parasites/μL were prepared by serially diluting the 10,000 parasites/μL stocks in 10 ng/μL of human DNA. Mock multiclonal infections of 3D7 and Dd2 were simulated at ratios 1:1, 5:1 and 10:1 using the 10,000 parasites/μL stocks of each strain at the appropriate volumes and then serially diluting the mixtures to 1,000, 100, and 10 parasites/μL. The 2:1 mixture was additionally evaluated at 1,000 parasites/μL only. All dilutions were carried out in 10 ng/μL of human DNA. We applied selective whole-genome amplification (SWGA) to all mock infections.

Clinical material was divided into three categories according to sample origin and DNA concentration as determined by the 4150 TapeStation System using the Genomic DNA ScreenTape Assay (Agilent Technologies, Santa Clara, CA, USA). The WB group (n = 95, Brazil) consisted of 1 mL WB extractions performed with the QIAamp DNA Blood Midi Kit (Qiagen), following the manufacturer's protocol, with DNA eluted in 200 μL. These samples showed a median parasitemia of 9,240 parasites/μL [IQR: 5,470–20,100] and a median library concentration of 5.0 ng/μL [IQR: 3.94–6.65].

The DBS group (n = 44, Mali) comprised DNA extracted from filter-paper bloodspots in a QIASymphony automated system as previously described.[27] Parasite density was then estimated with a *P. falciparum*-specific duplex qPCR assay developed for this study (as described below). The protocol was based on the previously published *pgmet* assay,[28] replacing the species-specific amplification of the *pfcytb* mitochondrial locus with the pan-genus *pgmet* target used in the original assay. For the 44 pre-treatment samples from Mali analyzed here, qPCR quantification was available for 42. Median peripheral parasite density estimated by qPCR was 5,958 parasites/μL [IQR: 1,828–12,596]. These samples presented a median library concentration of 2.7 ng/μL [IQR: 1.80–3.17].

The DBS-optimized (DBS-opt) group (n = 45, São Tomé and Príncipe) also consisted of DBS samples processed with QIAamp DNA Mini Kit (Qiagen), with total DNA eluted in 100 μL. These samples had a median parasitemia of 9,600 parasites/μL [IQR: 3,200–17,620] and a median library concentration of 0.5 ng/μL [IQR: 0.27–0.70]. After library construction, an additional purification and concentration step was performed to improve the final library yield prior to pooling. General characteristics of the clinical cohorts are summarized in Table S2.

An overview of the experimental workflow, from mock infection preparation through sequencing and variant detection, is shown in Figure 1.

Ethical approval was granted by the Human Research Ethics Committee of the Instituto René Rachou (CAAE: 45337921.0.0000.5091), IHMT-ITQB Ethics Committee (ref. 16.22), and the Swedish Ethical Review Authority (ref. 2017/499–32). Written informed consent was obtained from all participants prior to sample collection, in accordance with applicable ethical guidelines and regulations.

Parasite density quantification by qPCR

Parasite density in DBS samples from Mali was estimated using a modified duplex qPCR assay based on the previously described *pgmet* protocol.[28] The assay replaced the pan-genus target from the original assay with species-specific amplification of the *pfcytb* mitochondrial locus for *P. falciparum* quantification. Quantification was performed relative to the WHO International Standard for *P. falciparum* DNA using ΔCt normalization against the human *TUBB* reference gene, as previously described.[28] Detailed reaction conditions and cycling parameters are summarized in Table S10.

Validation of the duplex qPCR assay

The duplex qPCR assay was validated using 109 previously analyzed DBS-derived DNA samples from the MALACTRES project in western Kenya.[29] Samples included *P. falciparum*, *P. malariae*, and mixed infections previously quantified with the pan-genus qPCR assay. Correlation between assays was variable below 10 parasites mL^{-1} but showed strong agreement above this threshold, with parasite detection in one assay being highly predictive of detection in the other (Odds Ratio 26.47, 95% CI 8.128–111.5, $p < 0.0001$). Parasite density estimates were also strongly correlated between assays (Spearman's $\rho = 0.7065$, $p = 0.0011$). Based on this performance, the duplex assay was used for *P. falciparum* parasite density estimation in the present study.

METHOD DETAILS

Panel design

We developed a customized targeted NGS panel for the Agilent SureSelect XT HS2 target enrichment system, covering the full coding regions of 48 *P. falciparum* genes (Design ID 3488401). The 120-mer SureSelect probes were designed against the *P. falciparum* 3D7 reference genome (PlasmoDB release 63), and all target regions were covered with 3× tiling, resulting in 5,766 unique probes. These genes were selected based on classical molecular markers of antimalarial resistance and on recent studies implicating additional loci in drug response or parasite biology (Table S1). The panel consists of 46,719 probes with a total target size of 293 kb. The panel design is available free of charge upon request.

Selective whole-genome amplification (SWGA)

SWGA was performed to enrich *P. falciparum* DNA prior to library preparation, following the MalariaGEN protocol,[30] with bead-based purification performed subsequently. This approach was applied across all samples to standardize parasite DNA enrichment prior to sequencing, with particular benefits expected for low-input samples with high human DNA background, such as DBS. A pool of ten validated primers specific for the *P. falciparum* genome (5′-CGTTGCG-3′, 5′-TTTTTTCGC-3′, 5′-TCGTGCG-3′, 5′-CGTTTTTTTT-3′, 5′-TTTTTTTCGT-3′, 5′-CCGTTCG-3′, 5′-CGTTTCGT-3′, 5′-CGTTTCGC-3′, 5′-CGTTTTCG-3′, 5′-TCGTTCGT-3′) was used. These primers are designed to preferentially enrich parasite DNA in samples with high human DNA background, generating long amplicons suitable for downstream NGS library preparation. Amplification reactions (50 μL) contained ∼40 ng of DNA, Phi29 polymerase, and a primer pool with phosphorothioate modifications to prevent exonuclease degradation. Thermocycling followed a step-down isothermal program (35°C–30°C over 16 h), followed by enzyme inactivation. Amplified products were purified with AMPure XP magnetic beads (Beckman Coulter), using 20 μL of WGA product for most samples or 50 μL for the DBS-opt group to maximize recovery. DNA concentrations were measured with Qubit 4 fluorometry: Broad Range assays before and after WGA, and High Sensitivity assays prior to WGA in the DBS-opt group.

Library preparation and sequencing

Genomic libraries were prepared using the Magnis NGS Prep System (Agilent Technologies, Santa Clara, CA, USA), and the SureSelect XTHS2 DNA Reagent kit (Agilent Technologies, Santa Clara, CA, USA). For each sample, 200 ng of DNA in 14 μL was processed. The workflow included enzymatic fragmentation (15 min), seven cycles of pre-capture amplification, hybrid capture with the custom 48-gene panel, and twelve cycles of post-capture amplification. Library quality and fragment size distribution were assessed with the Agilent 4150 TapeStation System using the High Sensitivity D1000 ScreenTape Assay. Indexed libraries were pooled at 10 nM in 300 μL and sequenced on the Illumina NextSeq 2000 platform using the P1 flow cell kit (2 × 150 bp, 300 cycles) at the National Genomics Infrastructure (SciLifeLab, Sweden). For libraries originating from the DBS-opt group, a modified pooling strategy was used to ensure sufficient material for sequencing: libraries were first combined into a ∼2 nM pool in 300 μL and concentrated with AMPure XP magnetic beads (300 μL sample +540 μL beads, with the option to use a 1:1 ratio). The concentrated pool was eluted in 30 μL of Low TE buffer, checked with the 4150 TapeStation System using the High Sensitivity D1000 ScreenTape Assay, and then diluted to 10 nM for sequencing.

Bioinformatic processing

Raw reads were inspected with FastQC (v0.11.9) and summarized with MultiQC. Adapter/quality trimming was performed with Fastp using a Q20 threshold. To handle unique molecular identifiers (UMIs), the first and last six bases of each read were removed. Residual adapters were processed with Picard (FastqToSam, MarkIlluminaAdapters, SamToFastq). Cleaned reads were aligned to the *P. falciparum* 3D7 reference with BWA-MEM (v0.7.18). BAMs were sorted, and PCR duplicates were marked with GATK4 MarkDuplicates. Variants were called using two approaches for benchmarking: Mutect2 (GATK4) and HaplotypeCaller (GATK4). Mutect2 was selected for its ability to detect low-frequency alleles in mixed populations, a feature originally developed for somatic variant analyses[20] that is well suited for identifying minority parasite subpopulations in polyclonal malaria infections. HaplotypeCaller, a widely used caller in population genetics studies for germline variant analyses, was included to provide a comparative baseline for mixture quantification.[31] For Mutect2, variants were filtered with FilterMutectCalls, curated with SelectVariants, and standardized with bcftools (v1.20). For HaplotypeCaller, standard GATK hard-filtering practices were applied. All steps, from QC to VCF generation, were orchestrated by an in-house Nextflow pipeline (https://github.com/AntonioRezende/malvariant/tree/master). The workflow is implemented in Nextflow and integrates standardized nf-core modules and custom components, enabling reproducible, modular, and scalable execution across computing environments. Typical runtimes depend on sequencing depth and computational resources but are compatible with standard high-performance computing infrastructures.

Variant annotation and coverage analysis

Variants were annotated with SnpEff (v5.1). For mixture quantification, Dd2-fixed markers were defined as alleles present at ≥90% frequency in Dd2-pure controls. This threshold was selected to balance stringency with marker availability, which is also well above typical Illumina sequencing error rates (∼0.1–1%), ensuring that low-frequency technical noise does not affect marker classification. Experimental studies of *P. falciparum* drug resistance typically identify fixed alleles at or near 100% frequency under controlled

in vitro conditions,[26] but in genomic surveillance of field samples, absolute fixation is rarely observed due to technical variability in sequencing and alignment.[32] In these contexts, alleles above ~90–95% are often considered effectively fixed or predominant. Thus, our use of a ≥90% cutoff allowed reliable discrimination of Dd2-specific variants while retaining sufficient markers for downstream mixture analyses.

Coverage across the 48 coding targets was assessed with BEDTools using a custom BED file with the coding sequence (CDS) coordinates. For each sample-gene pair, base-level depth was aggregated to compute median depth and CDS breadth at depth cutoffs of 10×, 30× and 80×. A locus was considered successfully sequenced if ≥ 80% of its CDS bases met the respective depth cutoff. In addition to full-panel summaries, five canonical resistance genes (*pfcrt*, *pfmdr1*, *pfk13*, *pfdhfr*, and *pfdhps*) included in this panel were specifically analyzed, as they represent the most widely studied markers of antimalarial resistance and remain essential for surveillance. Summaries and figures were generated using custom Python/R code (Matplotlib v3.6.2; Seaborn v0.13.2; ggplot2).

Limit-of-detection modeling in mock infections

To quantify detection performance at increasing parasite DNA input, per-base depths were used to recompute CDS breadth for mock infections at 10, 100, and 1,000 parasites. For each depth threshold (10×, 30× and 80×), a logistic regression modeled the probability that a given gene in a given sample achieved success (CDS breadth ≥80%) as a function of $\log_{10}$ (parasite count). Replicates were analyzed individually, without averaging, to preserve within-condition variability. Pure controls (3D7-only and Dd2-only) were excluded from fitting because they yield near-certain success and do not inform the transition region that determines the detection limit. LoD95 was defined as the parasite count at which the fitted model predicts 95% success. Ninety-five percent confidence intervals for LoD95 were obtained by simulating the model's coefficient uncertainty and propagating it to LoD95 (i.e., model-based simulations).

Quantitative mixture analysis

To assess the accuracy of allele frequency estimation, observed variant allele frequencies (VAFs) were compared against theoretical expectations from 3D7:Dd2 mixtures (1:1, 2:1, 5:1, and 10:1 ratios). Dd2-fixed markers were defined as alleles with ≥90% frequency in Dd2-pure controls. Observed versus expected VAFs were evaluated per gene and dilution, and bias (median deviation), mean absolute error (MAE), and Spearman correlation coefficients (ρ) were calculated separately for each gene using all truth-set loci across all mixture ratios. Bias was defined as the median of (Observed VAF − Expected VAF) × 100, such that negative values indicate systematic underestimation of the expected frequency and positive values indicate overestimation. This gene-wise approach preserves the biological structure of the panel and prevents over-representation of genes containing more variants.

Within-host diversity analysis

To provide a complementary measure of infection complexity, within-host genetic diversity was estimated as the proportion of heterozygous loci per sample. Genotypes were classified as heterozygous when more than one allele was detected at a given locus based on genotype calls derived from the VCF files. The proportion of heterozygous loci was calculated as the number of heterozygous sites divided by the total number of successfully genotyped loci per sample. This metric was summarized across populations to compare patterns of within-host diversity. This approach provides a proxy for infection complexity but does not represent a formal estimate of multiplicity of infection (COI).

Truth set definition and qualitative evaluation

A truth set of high-confidence Dd2-specific variants was generated to serve as the reference for subsequent qualitative and quantitative analyses. Variant calls from Dd2-pure controls were processed separately for each caller, and loci were retained if they displayed a median variant allele frequency (VAF) ≥90% across Dd2 replicates, ensuring that only alleles consistently present at high frequency were considered. To exclude non-specific or background signals, loci showing a median VAF >1% in any 3D7-pure control were removed. The final truth set consisted of the intersection of loci meeting these criteria for both callers, yielding a conservative set of Dd2-specific variants used in all downstream assessments of detection and mixture estimation.

QUANTIFICATION AND STATISTICAL ANALYSIS

Coverage metrics, variant allele frequencies (VAFs), and within-host diversity estimates were calculated from VCF and depth files generated through the bioinformatic workflow. Logistic regression models were used to estimate the probability of successful target recovery across parasite densities, and LoD95 values were obtained through model-based simulations with 95% confidence intervals. Quantitative mixture analyses compared observed and expected VAFs using median bias, mean absolute error (MAE), and Spearman correlation coefficients. Summary statistics are presented as medians and interquartile ranges (IQR) unless otherwise specified. Bioinformatic and statistical analyses were performed using Python and R, and data visualization was conducted with Matplotlib, Seaborn, and ggplot2.

Cell Reports Methods, Volume *6*

Supplemental information

MalDeepSeq panel: A targeted ultra-deep sequencing approach to trace drug resistance markers in *Plasmodium falciparum*

Yanka E.A. R. Salazar, Antonio M. Rezende, Maria C.S. B. Puça, Sonja Lagström, Danielle Fletcher, Julian Muwanguzi-Karugaba, Colin J. Sutherland, Khalid B. Beshir, Maria E.P. Mascarenhas, Jaime Louzada, Joseli Oliveira-Ferreira, Abdoulaye Djimde, Dinora Lopes, Tais N. de Sousa, and José P. Gil

SUPPLEMENTAL FIGURES

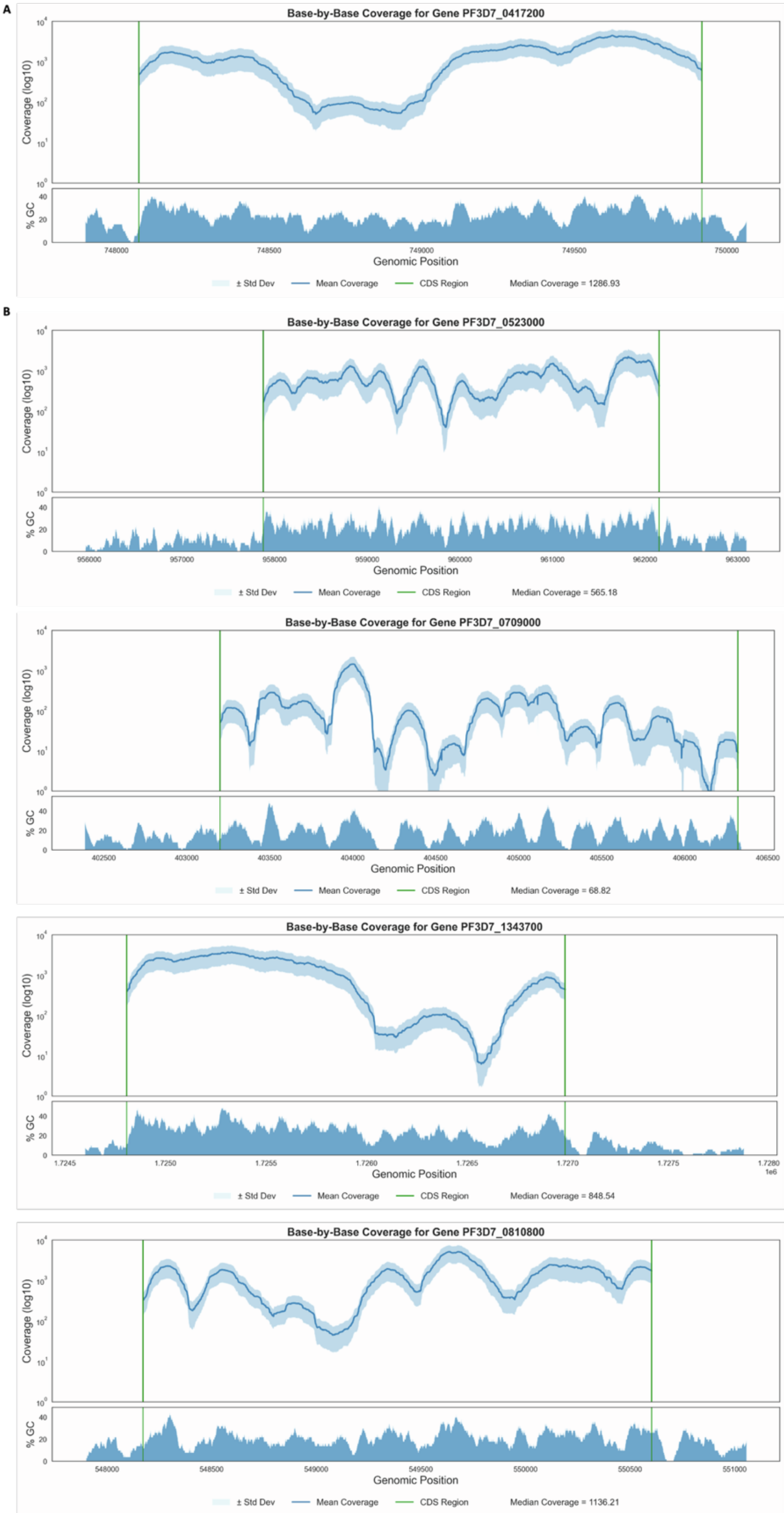

Figure S1. Per-base read depth and GC-content across target genes, related to Figure 2. Panels A–E show per-base read depth for each gene: A = *pfdhfr*, B = *pfmdr1*, C = *pfcrt*, D = *pfk13*, E = *pfdhps*. The solid line represents the mean read depth across samples at each genomic position, and the shaded band corresponds to ±1 SD. The CDS median read depth is reported in the legend as the median of the position-wise mean depth restricted to the CDS. Vertical lines mark CDS boundaries. The lower panel for each gene displays the GC-content (%) computed using a centered sliding window (50 bp), allowing visualization of GC variability across the region. X-axis: genomic coordinates fixed to the exact gene interval (start–end).

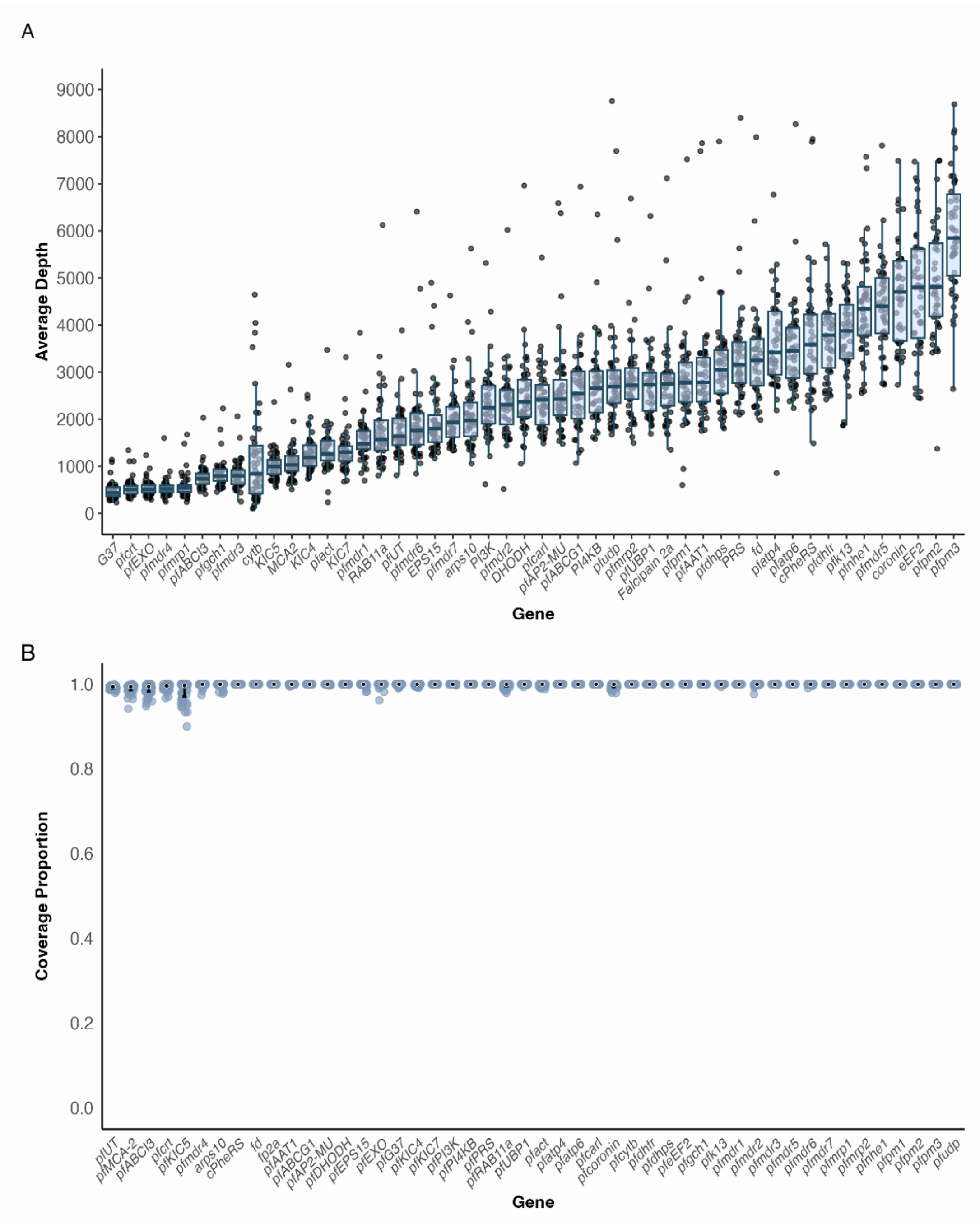

Figure S2. Sequencing depth and coverage proportion across the 48-gene panel in the WB group (Brazil), related to Figure 2. A) Average read depth per gene within the coding sequence (CDS), ordered by median depth. B) CDS coverage proportion (breadth) for the same genes. Overall performance was uniform, with consistently high depth and coverage across loci. The lowest median depth was observed for *G37* (430.96×).

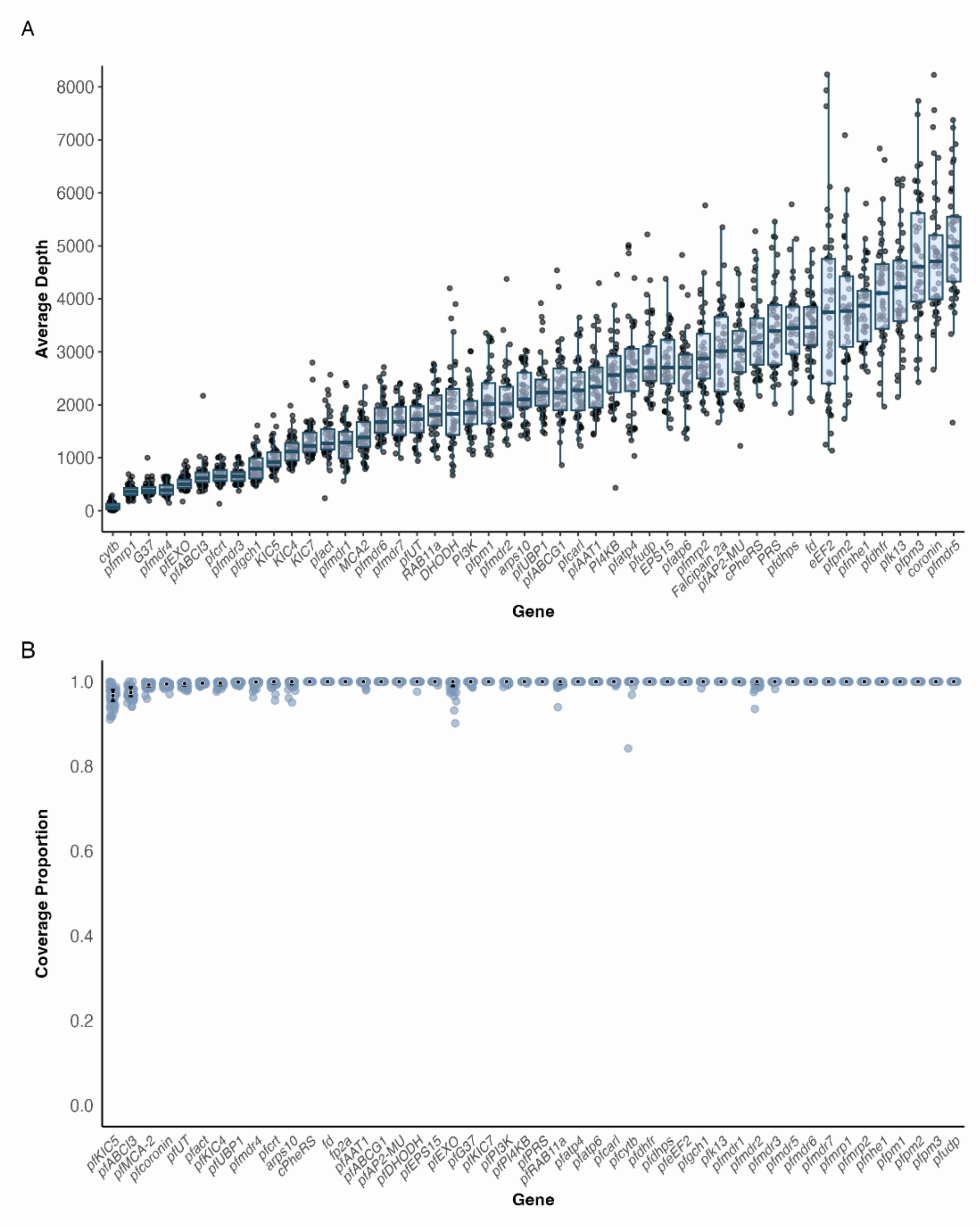

Figure S3. Sequencing depth and coverage proportion across the 48-gene panel in the DBS group (Mali), related to Figure 2. (A) Average CDS read depth and (B) CDS coverage proportion for the DBS group. The lowest median depth was 66.9× for *cytb*.

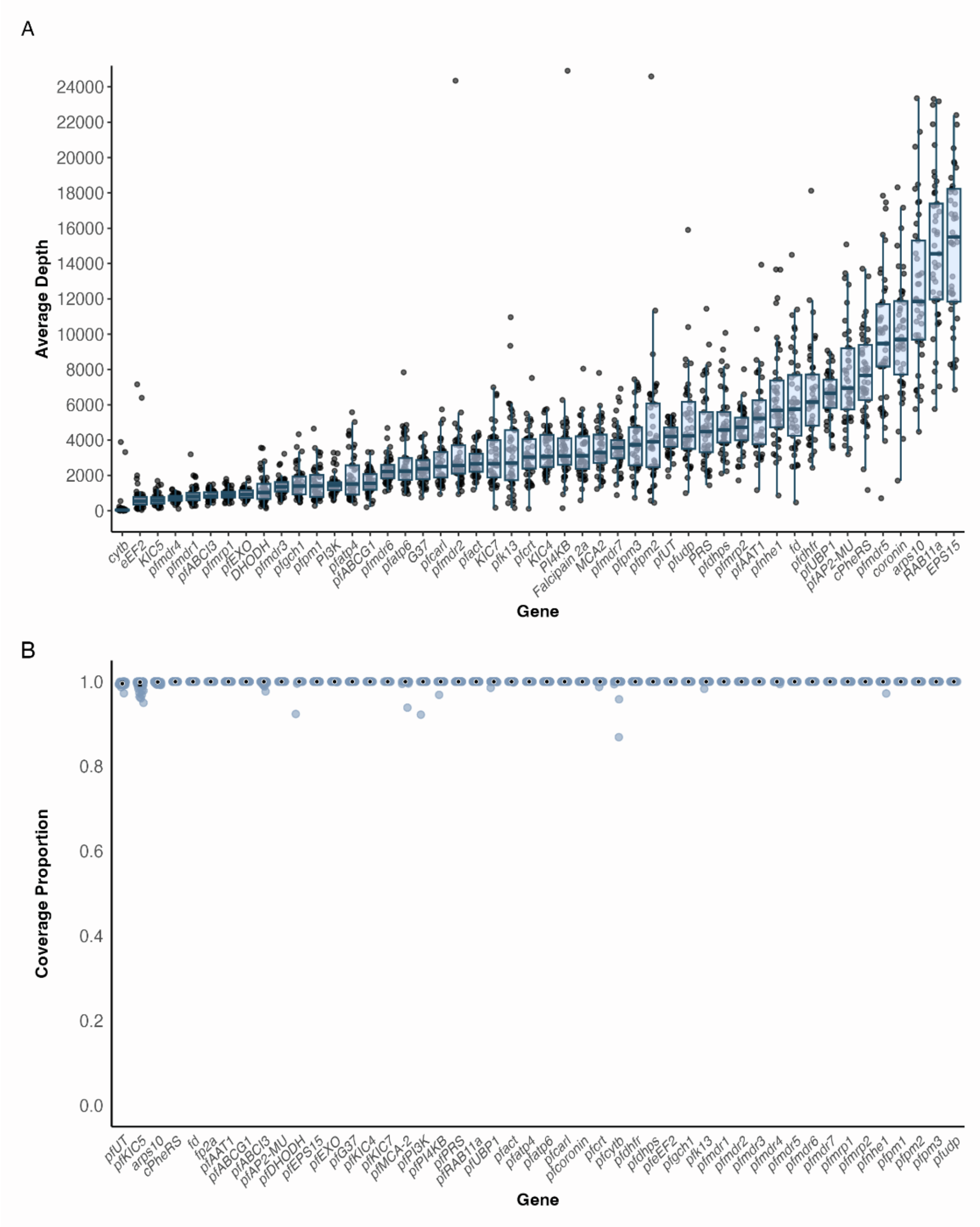

Figure S4. Sequencing depth and coverage proportion across the 48-gene panel in the DBS-opt group (São Tomé and Príncipe), related to Figure 2. A) Average CDS read depth and B) CDS coverage proportion. This subset exhibited the widest depth variability, with some loci reaching up to 24,000×, indicating efficient amplification despite limited starting material. The adapted workflow, including modified SWGA cleanup and pooling, likely contributed to maintaining robust coverage and overall high performance under low-DNA conditions. The lowest median depth was 36.3× for *cytb*.

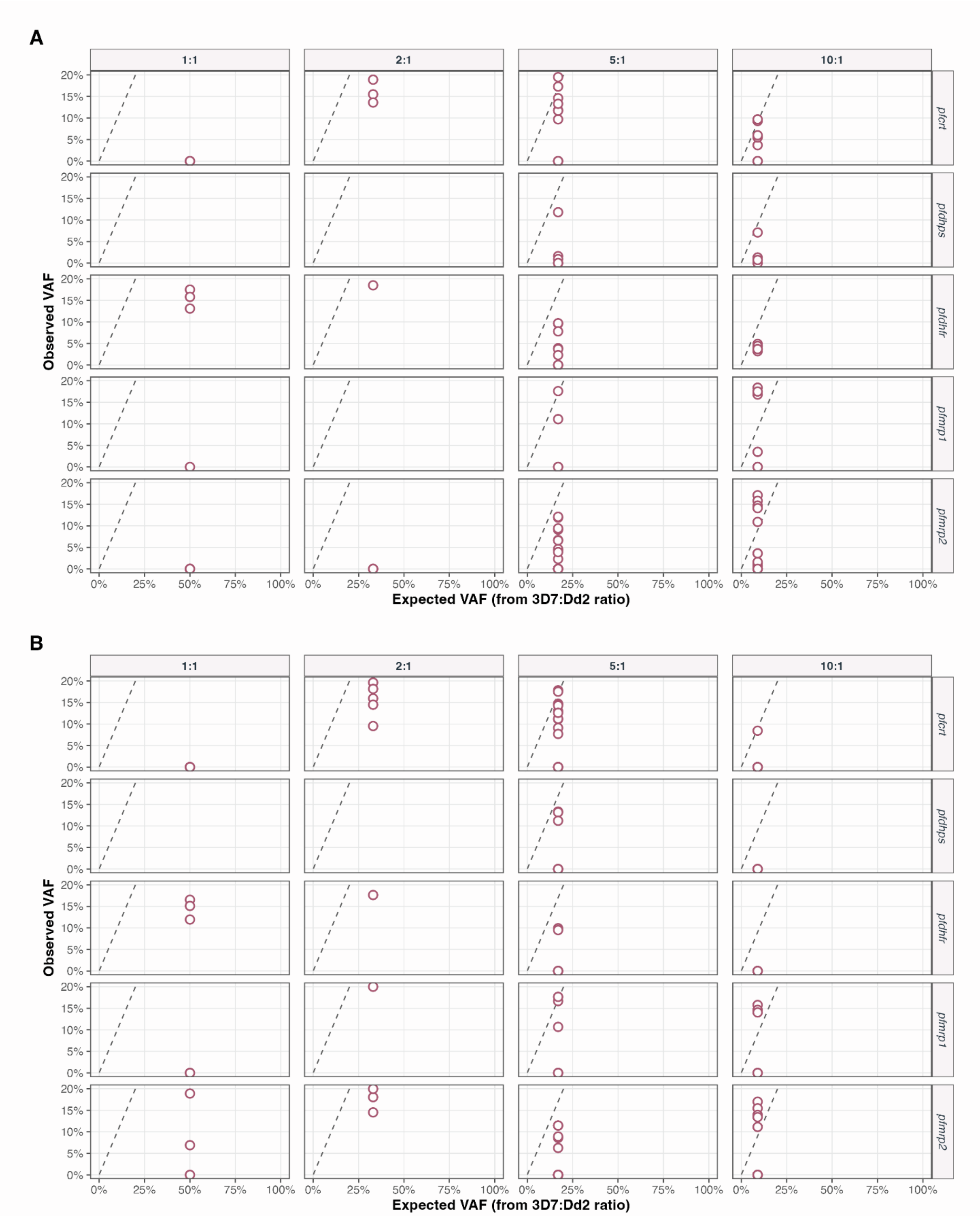

Figure S5. Zoomed view of observed versus expected variant allele frequencies (VAFs) in the low-frequency range (0–20%), related to Figure 3. (A) Mutect2 and (B) HaplotypeCaller. Points represent Dd2-specific loci across 3D7:Dd2 mixtures, stratified by gene and dilution ratio. The dashed line indicates the expected VAF. Compared with the full-range visualization, this zoom highlights reduced dispersion and improved agreement between observed and expected values in more asymmetric mixtures (5:1 and 10:1), while maintaining locus-specific variability.

SUPPLEMENTAL TABLES

Table S1. Genes included in the 48-target *Plasmodium falciparum* target enrichment panel related to the STAR Methods section.

Name	Gene size (nt)	Denomination	Chr Position (Start)	Chr Position (End)
pfcrt	3957	PF3D7_0709000	402385	406341
pfk13	3278	PF3D7_1343700	1724600	1727877
fpa2	3670	PF3D7_1115700	591136	594805
pfdhfr	2169	PF3D7_0417200	747897	750065
pfdhps	3162	PF3D7_0810800	547896	551057
pfcarl	6843	PF3D7_0321900	921776	928618
pfudp	2003	PF3D7_1113300	518688	520690
pfact	3854	PF3D7_1036800	1447919	1451772
cytb	1131	PF3D7_MIT02300	3492	4622
pfatp4	6137	PF3D7_1211900	527829	533965
pfatp6	5748	PF3D7_0106300	264487	270234
pfpm1	4162	PF3D7_1407900	287116	291277
pfpm2	3018	PF3D7_1408000	292244	295261
pfpm3	2419	PF3D7_1408100	296683	299101
pfEXO	3146	PF3D7_1362500	2502730	2505875
pfAAT1	4212	PF3D7_0629500	1213102	1217313
pfUT	15765	PF3D7_0704600	214949	230713
pfnhe1	8676	PF3D7_1303500	169481	178156
pfAP2-MU	3966	PF3D7_1218300	716700	720665
pfUBP1	12905	PF3D7_0104300	188420	201324
pfgch1	1872	PF3D7_1224000	974226	976097
pfmdr1	7141	PF3D7_0523000	955955	963095
pfmdr2	5674	PF3D7_1447900	1953474	1959147
pfmdr3	3335	PF3D7_1145500	1807031	1810365
pfmdr4	4821	PF3D7_0302600	141335	146155
pfmdr5	3457	PF3D7_1339900	1598111	1601567
pfmdr6	4359	PF3D7_1352100	2075535	2079893
pfmdr7	3247	PF3D7_1209900	448322	451568
pfmrp1	8128	PF3D7_0112200	463764	471891
pfmrp2	10802	PF3D7_1229100	1192185	1202986
pfABCI3	11052	PF3D7_0319700	820232	831283
pfABCG1	2344	PF3D7_1426500	1027682	1030025
fd	1256	PF3D7_1318100	748160	749415
arps10	2169	PF3D7_1460900	2480089	2482257
pfPI3K	6629	PF3D7_0515300	628784	635412
pfcPheRS	575	PF3D7_0109800	380254	382568
pfPI4KB	1559	PF3D7_0509800	409403	415758
pfDHODH	569	PF3D7_0603300	130191	132792
pfKIC7	599	PF3D7_0813000	643066	645158
pfEPS15	980	PF3D7_1025000	1045271	1051309
pfKIC5	1870	PF3D7_1138700	1525654	1532527
pfG37	349	PF3D7_1204400	208165	210893

pfPRS	746	PF3D7_1213800	589729	593777
pfKIC4	888	PF3D7_1246300	1925058	1929618
pfcoronin	602	PF3D7_1251200	2091731	2094720
pfRAB11a	216	PF3D7_1320600	845865	848770
pfMCA2	2020	PF3D7_1438400	1548072	1557053
pfeEF2	832	PF3D7_1451100	2089730	2094221

The 48 genes included in this panel were selected to represent three complementary categories: (i) well-established molecular markers of antimalarial resistance (e.g., *pfcrt, pfmdr1, pfk13, pfdhfr, pfdhps*); (ii) genes involved in drug transport, metabolism, or parasite fitness, potentially influencing treatment response; and (iii) emerging or putative loci identified in recent genomic, experimental, or functional studies. This design aims to capture both known resistance determinants and additional variation that may contribute to evolving resistance phenotypes.

Table S2. Summary of clinical sample cohorts included in the study related to the STAR Methods section.

Cohort	Study type	Country	Sample type	Parasitemia (parasites/µL), median [IQR]	Library concentration (ng/µL), median [IQR]	Treatment context
**WB**	Cross-sectional	Brazil	Whole blood	9,240 [5,470–20,100]	5.0 [3.94–6.65]	Artemether-Lumefantrine (first-line) with a single low-dose of Primaquine
**DBS**	Clinical Trial	Mali	Dried blood spots	5,958 [1,828–12,596]	2.7 [1.80–3.17]	Artemether-Lumefantrine (first-line); Ganaplacide-Lumefantrine (Phase-2b study)
**DBS-opt**	Cross-sectional	São Tomé and Príncipe	Dried blood spots	9,600 [3,200–17,620]	0.5 [0.27–0.70]	Artesunate-Amodiaquine (first-line)

WB, whole blood; DBS, dried blood spots; DBS-opt, optimized DBS protocol. Parasitemia values were estimated by qPCR for DBS samples and routine methods for WB samples, as described in the Methods.

Table S3. Panel performance at 10×, 30× and 80× by subset of samples related to the STAR Methods section.

Sample set	Median depth (all loci)[a]	Successful loci[b] (%) @10×	Successful loci (%) @30×	Successful loci (%) @80×
**WB group**	698	100.0	91.7	72.9
**DBS group**	550	97.9	85.4	58.3
**DBS-opt group**	860	100.0	95.8	85.4
**Mock infections (1000 parasites)**	385	93.8	78.1	51.0
**Mock infections (100 parasites)**	231	81.2	62.5	39.6
**Mock infections (10 parasites)**	49	52.1	35.4	22.9

[a] Median depth (all loci) is the median per-base coverage across all CDS positions, irrespective of pass/fail status

[b] Successful loci (%) is the percentage of target coding sequences (CDS) whose breadth (fraction of CDS bases covered) is ≥80% at the indicated depth cutoff (10×, 30×, or 80×).

Table S4. Logistic model of CDS success versus parasite input in mock infections related to the STAR Methods section.

Depth threshold	Intercept (b0)	Slope (b1)	LoD95 (95% CI)
**10×**	-1.32	1.44	905 (448–2,609)
**30×**	-1.41	0.97	32,946 (8,751–322,131)
**80×**	-1.78	0.67	11,794,634 (512,015–14,846,122)

N = 432 gene–sample rows (9 mock mixtures × 48 loci). LoD95 and 95% confidence intervals were estimated from a logistic regression of success (CDS breadth ≥80%) versus $\log_{10}$(parasite input), with 10,000 parametric draws from the coefficient covariance matrix to generate percentile intervals. LoD95 represents the total parasite count at which the fitted model predicts 95% success, reflecting analytical sensitivity rather than clinical parasitemia.

Table S5. Comparison of median sequencing depth (IQR) across *Plasmodium falciparum* target genes obtained from WB, DBS and DBS-opt samples, related to Figure 2.

Gene	WB group	DBS group	DBS-opt group
pfDHODH	2367.60 (2049.90–2839.69)	1825.88 (1431.58–2302.88)	1032.71 (664.58–1528.57)
pfEPS15	1800.66 (1512.83–2084.57)	2705.03 (2398.43–3230.72)	15498.80 (11837.67–18224.77)
fpa2	2748.27 (2284.55–2970.85)	3013.64 (2249.68–3658.70)	3113.57 (2369.54–4218.84)
pfG37	430.96 (359.98–557.19)	376.43 (329.54–448.33)	2379.16 (1779.87–2873.33)
pfKIC4	1187.34 (1003.34–1448.35)	1118.72 (947.12–1278.08)	3068.52 (2478.25–4303.29)
pfKIC5	994.39 (827.95–1137.66)	918.91 (831.05–1105.13)	597.45 (412.88–769.23)
pfKIC7	1300.46 (1111.42–1428.15)	1224.19 (1109.43–1476.53)	2664.10 (1889.44–3999.49)
pfMCA2	1026.59 (899.55–1218.96)	1382.31 (1206.02–1676.50)	3301.97 (2706.96–4314.90)
PI3K	2242.01 (1901.80–2715.66)	1851.88 (1634.44–2072.51)	1444.24 (1163.71–1616.18)
PI4KB	2661.78 (2142.59–3024.74)	2560.37 (2237.22–2926.81)	3100.47 (2595.26–4111.88)
PRS	3156.36 (2766.03–3636.64)	3395.95 (2754.61–3884.98)	4476.81 (3312.15–5581.88)
pfRAB11a	1567.26 (1335.83–1976.21)	1808.39 (1605.46–2181.53)	14554.05 (11968.63–17398.65)
arps10	1973.41 (1634.19–2355.64)	2104.18 (1956.40–2609.10)	11852.34 (9691.44–15297.82)
pfcPheRS	3582.49 (2962.83–4227.09)	3176.70 (2756.38–3635.00)	7656.09 (6270.50–9389.10)
pfcoronin	4700.10 (3659.60–5362.01)	4705.37 (3991.43–5202.65)	9699.64 (7718.10–11874.49)
cytb	840.74 (422.32–1438.61)	66.95 (30.14–134.04)	36.36 (20.15–71.44)
pfeEF2	4799.27 (3723.91–5617.53)	3745.10 (2401.78–4752.73)	538.92 (340.32–781.91)
fd	3251.35 (2710.52–3705.70)	3466.15 (3121.47–3845.39)	5754.33 (4246.29–7686.74)
pfAAT1	2783.61 (2372.01–3306.08)	2339.34 (1947.06–2707.46)	5227.25 (3758.77–6253.67)
pfABCG1	2545.43 (2012.14–3013.93)	2236.17 (1899.98–2685.72)	1555.14 (1187.27–2080.02)
pfABCI3	734.69 (623.15–846.98)	618.92 (538.32–725.66)	835.98 (729.73–1043.85)
pfAP2-MU	2428.31 (2083.36–2838.77)	3027.69 (2615.38–3397.81)	6947.46 (5743.98–9203.49)
pfEXO	510.85 (442.26–601.16)	503.20 (428.24–589.13)	920.14 (730.80–1149.21)
pfUBP1	2730.90 (2178.07–2984.21)	2232.20 (1985.65–2476.21)	6652.93 (5816.01–7432.69)
pfUT	1635.11 (1450.05–2021.21)	1726.70 (1476.33–1972.77)	4205.42 (3613.09–4713.58)
pfact	1264.10 (1122.08–1542.93)	1271.77 (1157.92–1538.49)	2660.29 (2206.22–3226.40)
pfatp4	3416.62 (2943.24–4288.41)	2645.60 (2258.32–3057.08)	1516.46 (909.96–2576.70)
pfatp6	3450.23 (2868.80–3941.83)	2706.05 (2252.27–2947.45)	2240.22 (1751.59–2980.97)
pfcarl	2415.58 (1884.32–2731.22)	2273.14 (1892.75–2615.81)	2499.13 (1879.33–3326.30)
pfcrt	504.95 (420.41–586.64)	645.54 (560.26–771.90)	3034.51 (2388.08–4063.86)
pfdhfr	3781.89 (3091.08–4240.46)	4108.51 (3433.83–4652.45)	6151.37 (4813.23–7718.97)
pfdhps	3047.67 (2544.67–3465.39)	3450.77 (2961.80–3854.90)	4569.59 (3859.98–5595.46)
pfgch1	796.15 (690.17–942.66)	792.40 (606.62–994.86)	1395.71 (920.22–1944.12)
pfk13	3873.47 (3283.36–4430.12)	4220.91 (3579.62–4721.01)	2699.21 (1725.46–4564.03)
pfmdr1	1476.22 (1359.83–1752.97)	1287.43 (986.97–1494.63)	803.28 (596.47–1009.81)
pfmdr2	2308.95 (1893.95–2628.70)	2022.73 (1765.90–2341.00)	2562.17 (2103.99–3710.30)
pfmdr3	800.15 (616.05–923.58)	648.91 (560.55–728.45)	1350.09 (1091.37–1623.84)
pfmdr4	518.32 (447.93–592.21)	388.15 (304.30–485.23)	704.20 (566.31–849.79)

pfmdr5	4400.85 (3823.06–4996.70)	4986.89 (4326.75–5548.00)	9464.73 (8165.24–11702.69)
pfmdr6	1758.14 (1458.25–2130.92)	1670.88 (1469.95–1938.16)	2223.21 (1830.14–2598.75)
pfmdr7	1932.09 (1614.83–2262.04)	1680.66 (1430.35–1962.87)	3570.18 (2970.61–3999.37)
pfmrp1	541.35 (454.33–597.08)	360.66 (296.52–433.15)	903.48 (750.96–1081.04)
pfmrp2	2720.11 (2425.11–3085.62)	2871.49 (2493.97–3341.49)	4727.88 (3989.99–5274.29)
pfnhe1	4339.80 (3778.31–4813.48)	3870.77 (3194.55–4154.62)	5681.82 (4722.25–7385.13)
pfpm1	2775.97 (2362.19–3204.55)	2017.80 (1647.09–2410.16)	1408.63 (776.27–2028.62)
pfpm2	4808.87 (4182.86–5734.76)	3769.97 (3095.04–4428.22)	3903.72 (2466.85–6081.70)
pfpm3	5847.36 (5046.01–6776.87)	4609.65 (3946.37–5619.44)	3738.68 (2570.14–4734.04)
pfudp	2691.28 (2342.42–3042.44)	2696.75 (2447.87–3102.28)	4240.64 (3503.83–6162.77)

Table S6. Global mixture quantification metrics by gene for Mutect2 and HaplotypeCaller, related to Figure 3.

Gene	Caller	Spearman ρ	Spearman p-value	Global bias (pp)	Global MAE (pp)	Wilcoxon p
pfcrt	HaplotypeCaller	0.583	3.73e-06	−9.00	9.00	6.69e-10
	Mutect2	0.470	1.13e-03	−9.00	9.00	3.92e-07
pfdhfr	HaplotypeCaller	0.863	6.87e-09	−15.37	16.91	2.60e-04
	Mutect2	0.771	2.54e-06	−9.20	13.30	1.45e-03
pfdhps	HaplotypeCaller	0.875	2.40e-09	−9.00	12.73	1.27e-02
	Mutect2	0.790	9.61e-05	−9.55	14.15	4.50e-02
pfmrp1	HaplotypeCaller	0.624	5.01e-04	+0.65	9.00	7.64e-01
	Mutect2	0.719	2.38e-05	+3.10	9.00	5.40e-01
pfmrp2	HaplotypeCaller	0.771	2.28e-15	−9.00	15.05	1.96e-03
	Mutect2	0.377	1.09e-03	−9.65	17.00	2.55e-04

Spearman ρ = Spearman rank correlation coefficient between expected and observed variant allele frequencies (VAFs). Spearman p-value = significance of correlation. Global bias (pp) = median difference in percentage points (observed – expected VAF). Global MAE (pp) = mean absolute error in percentage points. Global p_Wilcoxon = p-value from paired Wilcoxon signed-rank test across all dilutions. Negative bias values indicate systematic underestimation of the resistant allele frequency relative to expectation.

Table S7. Mixture quantification metrics by dilution ratio for Mutect2 and HaplotypeCaller, related to Figure 3.

Gene	Caller	Ratio (3D7:Dd2)	Bias (pp)	MAE (pp)	Wilcoxon p
pfcrt	Mutect2	1:1	-14,1	14,1	0.012
		2:1	-14,1	14,1	0.062
		5:1	-5,2	5,3	0.770
		10:1	-3,45	3,45	0.014
	HaplotypeCaller	1:1	-13,31	13,31	2.54×10^{-5}
		2:1	-15,96	15,96	0.007
		5:1	-5,84	5,84	0.001
		10:1	-9	9	0.0001
pfdhfr	Mutect2	1:1	-18,7	20	0.129
		2:1	-8,9	8,9	0.250
		5:1	-13,3	13,3	0.008
		10:1	-4,95	4,95	0.035
	HaplotypeCaller	1:1	-19,32	21,05	0.097
		2:1	-10,23	10,23	0.250
		5:1	-17	17	0.006
		10:1	-9	9	0.019
pfdhps	Mutect2	1:1	-11,5	17,4	1.000
		2:1	1,55	1,55	1.000
		5:1	-16,54	16,54	0.034
		10:1	-7,99	7,99	0.125
	HaplotypeCaller	1:1	-12,73	15,4	0.910
		2:1	1,98	1,98	0.500
		5:1	-17	17	0.007
		10:1	-9	9	0.019
pfmrp1	Mutect2	1:1	15,7	16,7	0.041
		2:1	-2,6	2,6	0.083
		5:1	0,6	8	0.221
		10:1	1,15	8,75	0.318
	HaplotypeCaller	1:1	4,55	30	0.006
		2:1	-3,83	3,83	0.012
		5:1	-0,33	7,56	0.044
		10:1	-1,98	7,89	0.057
pfmrp2	Mutect2	1:1	-14,75	24,4	0.029
		2:1	-9,2	9,2	0.071
		5:1	-17	17	0.165
		10:1	-7,55	7,9	0.228
	HaplotypeCaller	1:1	10,37	20,17	0.009
		2:1	-9,92	9,92	0.016
		5:1	-17	17	0.038
		10:1	-9	9	0.064

Ratio (3D7:Dd2) denotes the input proportion of 3D7 and Dd2 used to generate the mock mixtures and define the expected VAF for each dilution. Bias (pp) represents the median difference in percentage points between observed and expected VAF (observed – expected). MAE (pp) corresponds to the median absolute deviation in percentage points from the expected VAF. Wilcoxon p indicates the p-value from a paired Wilcoxon signed-rank test evaluating the observed–expected VAF differences for each gene–caller–dilution combination. Negative Bias values reflect systematic underestimation of the Dd2 allele frequency, whereas positive values reflect overestimation.

Table S8. Quantitative performance metrics by gene, parasite density, and mixture ratio, related to Figure 3.

Caller	Gene	Parasitemia (p/µL)	1:1 (Bias/MAE)	2:1 (Bias/MAE)	5:1 (Bias/MAE)	10:1 (Bias/MAE)	Spearman ρ
**HaplotypeCaller**	*pfcrt*	10	-50.00/50.00	NA	-17.00/17.00	NA	NA
**HaplotypeCaller**	*pfcrt*	100	-7.04/7.04	NA	-3.56/3.56	-9.00/9.00	0.96*
**HaplotypeCaller**	*pfcrt*	1000	-10.26/10.26	-15.96/15.96	-3.15/4.62	-9.00/9.00	0.90*
**HaplotypeCaller**	*pfdhfr*	10	16.67/16.67	NA	-17.00/17.00	NA	0.93*
**HaplotypeCaller**	*pfdhfr*	100	-34.91/34.91	NA	-17.00/17.00	-9.00/9.00	0.84*
**HaplotypeCaller**	*pfdhfr*	1000	-19.32/19.32	-10.23/10.23	-7.51/7.51	-9.00/9.00	0.91*
**HaplotypeCaller**	*pfdhps*	10	41.61/41.61	NA	-17.00/17.00	NA	0.93*
**HaplotypeCaller**	*pfdhps*	100	-15.40/15.40	NA	-17.00/17.00	-9.00/9.00	0.84*
**HaplotypeCaller**	*pfdhps*	1000	-12.73/12.73	1.98/1.98	-3.84/3.84	-9.00/9.00	0.91*
**HaplotypeCaller**	*pfmrp1*	10	-50.00/50.00	NA	-17.00/17.00	NA	0.45
**HaplotypeCaller**	*pfmrp1*	100	30.00/30.00	NA	-0.33/0.65	-9.00/9.00	0.96*
**HaplotypeCaller**	*pfmrp1*	1000	1.52/3.01	-3.83/3.83	7.56/7.56	5.63/5.63	0.91*
**HaplotypeCaller**	*pfmrp2*	10	22.82/30.14	NA	-17.00/17.00	NA	0.80*
**HaplotypeCaller**	*pfmrp2*	100	18.01/18.01	NA	-17.00/17.00	-9.00/9.00	0.84*
**HaplotypeCaller**	*pfmrp2*	1000	-19.44/19.44	-9.92/9.92	-8.25/8.25	3.27/7.22	0.71*
**Mutect2**	*pfcrt*	10	-50.00/50.00	NA	-17.00/17.00	NA	NA
**Mutect2**	*pfcrt*	100	-9.10/9.10	NA	-2.40/3.10	-3.50/3.50	0.95*
**Mutect2**	*pfcrt*	1000	-12.30/12.30	-14.10/14.10	0.30/5.20	-3.40/3.40	0.91*
**Mutect2**	*pfdhfr*	10	17.70/17.70	NA	-17.00/17.00	NA	0.93*
**Mutect2**	*pfdhfr*	100	-34.20/34.20	NA	-13.30/13.30	-5.30/5.30	0.64
**Mutect2**	*pfdhfr*	1000	-18.70/18.70	-8.90/8.90	-7.40/7.40	-4.60/4.60	0.95*
**Mutect2**	*pfdhps*	10	41.45/41.45	NA	-17.00/17.00	NA	0.94
**Mutect2**	*pfdhps*	100	-17.40/17.40	NA	-15.74/15.74	-7.99/7.99	0.84*
**Mutect2**	*pfdhps*	1000	-11.50/11.50	1.55/1.55	-11.10/11.10	-5.45/5.45	0.91*
**Mutect2**	*pfmrp1*	10	16.70/40.00	NA	-17.00/17.00	NA	0.69
**Mutect2**	*pfmrp1*	100	28.60/28.60	NA	0.60/5.90	-9.00/9.00	0.95*
**Mutect2**	*pfmrp1*	1000	3.10/3.10	-2.60/2.60	6.60/6.60	8.50/8.50	0.89*
**Mutect2**	*pfmrp2*	10	20.40/29.50	NA	-17.00/17.00	NA	0.69*
**Mutect2**	*pfmrp2*	100	19.35/22.90	NA	-15.85/15.85	-8.65/8.65	0.38
**Mutect2**	*pfmrp2*	1000	-20.90/20.90	-9.20/9.20	-9.10/9.10	3.50/6.25	0.27

Bias and MAE are expressed in percentage points (pp) and represent the median deviation between observed and expected variant allele frequencies and the median absolute error, respectively. Values are shown by gene, variant caller, parasite density, and mixture ratio (3D7:Dd2). Spearman's rho (ρ) reflects the monotonic agreement between observed and expected VAFs across mixture ratios for each gene and parasitemia level. Asterisks (*) indicate statistically significant correlations ($p < 0.05$). NA indicates unavailable estimates.

Table S9. Within-host heterozygosity across Mali, São Tomé and Príncipe and Brazil, related to subclonal variation in the Results section.

Population	n	Heterozygosity, median (IQR)	Heterozygosity, mean (sd)
**Mali**	44	0.0292 (0.0241–0.0335)	0.0286 (0.0156)
**São Tomé and Príncipe**	45	0.0194 (0.0152–0.0258)	0.0217 (0.0078)
**Brazil**	95	0.0158 (0.0119–0.0204)	0.0163 (0.0063)

Within-host heterozygosity was estimated as the proportion of heterozygous loci per sample. Values are presented as the number of samples (n), median with interquartile range (IQR), mean, and standard deviation (sd). Heterozygosity was calculated as the ratio of heterozygous genotype calls to the total number of successfully called loci per sample.

Table S10. Summary of duplex *pfcytb* qPCR assay conditions related to the STAR Methods section.

Category	Description
**Assay format**	Duplex hydrolysis probe qPCR targeting *P. falciparum pfcytb* and human *htubb*
***pfcytb* primers**	F: 5′-TGC AATT GTT AAA AAC TGT TCC AAG TA-3′ R: 5′-AGT TGT TAA ACT TCT TTG TTC TGC T-3′
***pfcytb* probe**	5′-(FAM)-CCA GCT GGT TTA GTA ATT GTA TTA TTA TCA-(BHQ1)-3′
***htubb* primers**	F: 5′-AAG GAG GTC GAT GAG CAG AT-3′ R: 5′-GCT GTC TTG ACA TTG TTG GG-3′
***htubb* probe**	5′-(Cy5)-TTA ACG TGC AGA ACA AGA ACA GCA GCT-(BHQ2)-3′
**Reaction conditions**	25 µL final volume containing 5 µL genomic DNA, 1× NH_4 buffer, 5.5 mM $MgCl_2$, 0.3 mM dNTPs, 0.3 µM primers, 0.2 µM probes, and 1 U Taq polymerase
**Cycling conditions**	95 °C for 3 min, followed by 40 cycles of 95 °C for 15 s, 56 °C for 15 s, and 72 °C for 30 s
**Quantification approach**	ΔCt normalization against *htubb* and ΔΔCt-based estimation relative to the WHO International Standard for *P. falciparum* DNA (49,000 parasites mL^{-1})
